# Visual experience induces 4–8 Hz synchrony between V1 and higher-order visual areas

**DOI:** 10.1016/j.celrep.2023.113482

**Published:** 2023-11-22

**Authors:** Yu Tang, Catherine Gervais, Rylann Moffitt, Sanghamitra Nareddula, Michael Zimmermann, Yididiya Y. Nadew, Christopher J. Quinn, Violeta Saldarriaga, Paige Edens, Alexander A. Chubykin

**Affiliations:** 1 Department of Biological Sciences, Purdue Institute for Integrative Neuroscience, Purdue Autism Research Center, Purdue University, West Lafayette, IN 47907, USA; 2 Department of Computer Sciences, Iowa State University, Ames, IA 50011, USA; 3 Lead contact

## Abstract

Visual perceptual experience induces persistent 4–8 Hz oscillations in the mouse primary visual cortex (V1), encoding visual familiarity. Recent studies suggest that higher-order visual areas (HVAs) are functionally specialized and segregated into information streams processing distinct visual features. However, whether visual memories are processed and stored within the distinct streams is not understood. We report here that V1 and lateromedial (LM), but not V1 and anterolateral, become more phase synchronized in 4–8 Hz after the entrainment of visual stimulus that maximally induces responses in LM. Directed information analysis reveals changes in the top-down functional connectivity between V1 and HVAs. Optogenetic inactivation of LM reduces post-stimulus oscillation peaks in V1 and impairs visual discrimination behavior. Our results demonstrate that 4–8 Hz familiarity-evoked oscillations are specific for the distinct visual features and are present in the corresponding HVAs, where they may be used for the inter-areal communication with V1 during memory-related behaviors.

## In brief

We demonstrate that visual familiarity induces 4–8 Hz synchrony between higher-order visual areas (HVAs) and V1 in mice. Directed information analysis revealed changes in the functional connectivity from HVAs to V1. Optogenetic inactivation of LM during the delay period after the visual stimulus reduced the 4–8 Hz oscillation amplitude in V1 and impaired visual discrimination behavior.

## Graphical abstract

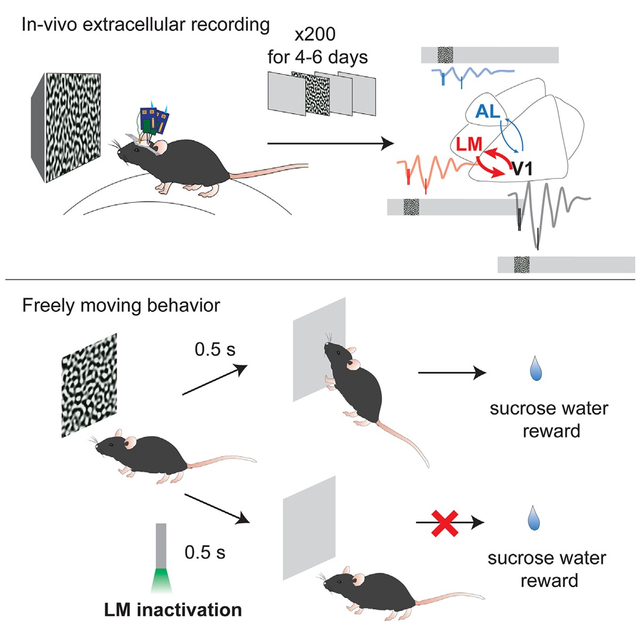

## INTRODUCTION

Visual processing in mice is performed by the primary visual cortex (V1) and higher-order visual areas (HVAs). Previous reports suggest that the HVAs are functionally specialized.^1–4^ The inter-areal connections between visual cortical areas are segregated^5,6^ and contribute to visual feature selectivity in HVAs, receptive field properties, and responses to visual illusions in V1.^7–11^ The HVAs form two anatomically and functionally segregated information-processing streams.^12–15^ The lateromedial (LM) area and the anterolateral (AL) area are considered gateways for these two streams.^12,13^ Recent studies demonstrated that even simple visual feature-processing in mice was distributed in both V1 and HVAs. Optogenetic suppression of LM or AL impaired the mouse’s ability to discriminate orientations and contrasts, while suppressing the posteromedial (PM) area had no effect.^16^ Inhibiting feedback from LM to V1 also partially impaired the detection of contrast changes.^17^

Previous work suggests that neuronal responses in the mouse V1 can report not only visual information but also visually cued non-sensory information during and after the visual stimulation. Neurons in V1 could become preferentially responsive to the rewarded visual stimulus, demonstrating the largest response when mice continued to lick to get a water reward.^18^ The visually cued non-sensory information could also be encoded in persistent neuronal activity lasting to the time of reward after the visual stimulation.^19–21^ Visual perceptual experience alone without behavioral outcome could also induce different forms of neuronal activity that encoded non-sensory information, such as stimulus-selective response potentiation and persistent 4–8 Hz oscillatory activity.^22–25^ Interestingly, the persistent 4–8 Hz oscillations were specific to the familiar visual stimulus and its spatial frequency (SF).^22^ What are the relevant neural circuits underlying these familiarity-induced oscillations? We have previously shown that, in V1, intracortical projections from layer 5 excitatory neurons onto the superficial layers were strengthened in mice after the visual experience.^23,26^ However, whether these familiarity-induced oscillations are present in other brain areas is not known. Theta oscillations have been described previously in primates’ V1 and V4, where they were coherent, and this coherency was associated with inter-areal communication.^27,28^ Low-frequency oscillations are well positioned for mediating long-range communication because of their long excitability duration.^29–34^ Thus, we hypothesized that the visual familiarity-induced 4–8 Hz oscillations might represent the mechanism of inter-areal synchronization between V1 and HVAs required for processing the familiar content.

To test this hypothesis, we first examined whether visual familiarity-induced oscillations were present in HVAs, and then investigated whether V1 and HVA synchrony changed following the visual experience by recording simultaneous activity from V1 and LM or from V1 and AL using silicon probes. Here, we report the expression of familiarity-evoked 4–8 Hz oscillations in V1 and superficial layers in LM and AL. Next, based on our previous discovery of the specificity of 4–8 Hz oscillations for the SF of the familiar stimulus and the selectivity of LM and AL areas for the distinct combinations of spatial and temporal frequencies (TFs) of the stimulus, we reasoned that familiarity-evoked 4–8 Hz oscillations could synchronize either LM or AL selectively with V1 depending on the visual features. Indeed, we discovered enhanced 4–8 Hz phase synchronization between V1 and LM, but not between V1 and AL, after the entrainment of the SF and the TF that maximally induced response in LM. Enhanced phase synchronization could also be induced between V1 and AL after the entrainment of SF and TF that maximally induced response in AL. The 4–8 Hz phase synchronization increase was most strongly expressed in response to the entrained SF and TF, suggesting that this synchrony might be dependent on the neural circuits selective for the entrained SF and TF. Optogenetic inactivation of LM reduced post-stimulus oscillations in V1, indicating top-down modulation of the familiarity-evoked persistent oscillations in V1. Finally, inactivating the familiarity-evoked persistent oscillations in LM post-stimulus impaired behavior in the visual discrimination task, suggesting the role of familiarity-evoked oscillations in V1 and HVAs for memory-related behaviors.

## RESULTS

**Figure 1.**
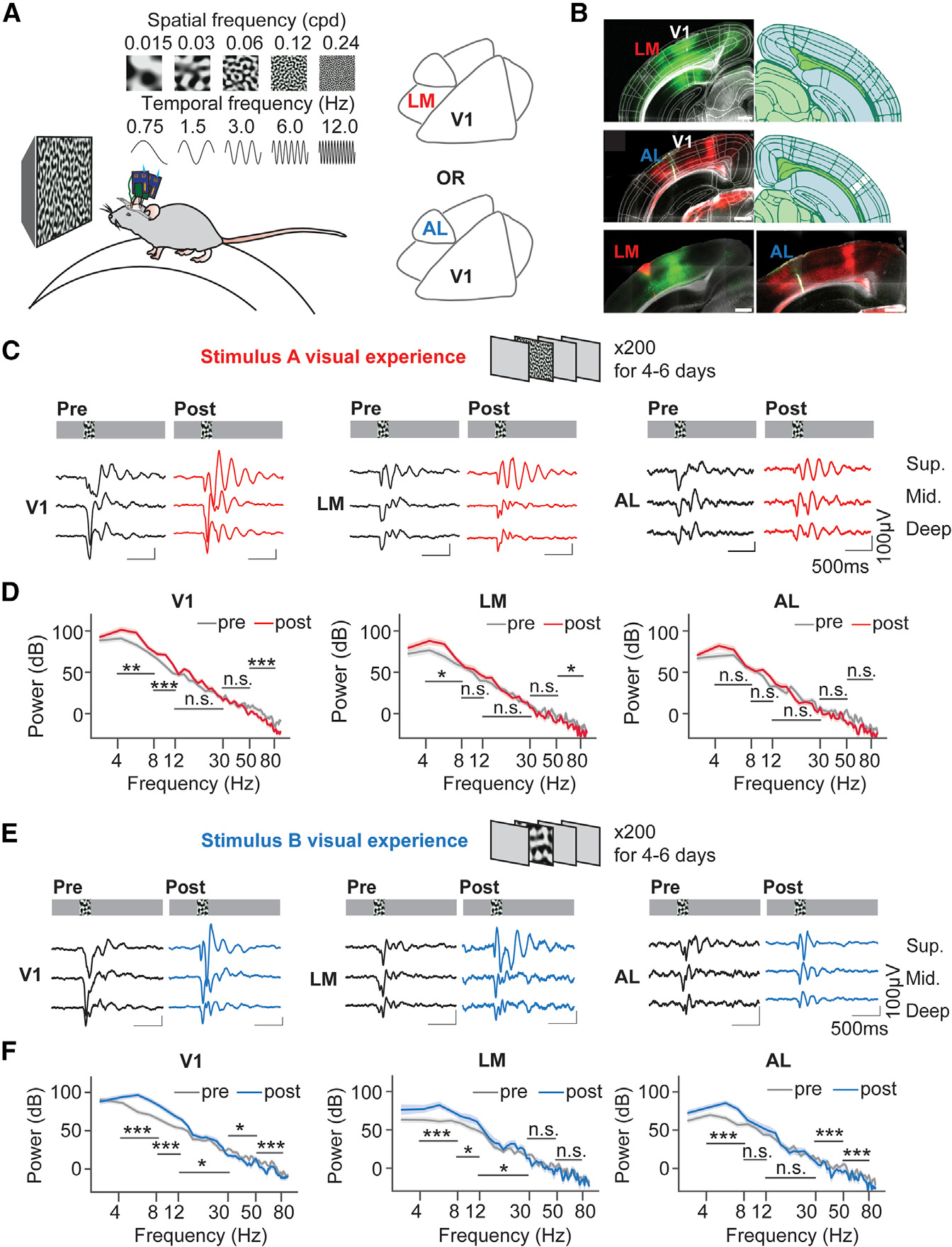
4–8 Hz oscillations were enhanced in superficial layers of V1 and higher-order visual areas after the visual experience (A) The experimental setup. Extracellular activity inV1 and in a higher-order visual area (LM or AL) was recorded simultaneously in a head-fixed awake mouse running on a treadmill. Twenty-five pink noise stimuli that were spatially filtered around five spatial frequencies (SFs) with pixel light intensity changed at five temporal frequencies (TFs) were presented. (B) Representative histology showed fluorescence labeling of V1 and higher-order visual areas (green, LM; red, AL). The fluorescence in LM or AL was achieved by injecting either retrograde or anterograde AAV in V1. The brain atlas was mapped to the brain slice using Allen CCFv3. Probe tracks were indicated by DiD or DiO dye. A representative brain slice showed the probe track in LM in red, and another slice showed the probe track in AL in green. Scale bars, 500 μm. (C) The visual stimulus with SF and TF that induced the largest response in LM was used in the visual experience (SF = 0.12 cpd, TF = 0.75 Hz). The same stimulus was presented for 200 repeats per day for 4–6 days. Averaged LFP traces across mice before and after the visual experience are shown below. V1: n_pre_ = 60 LFPs, 20 mice, n_post_ = 60 LFPs, 20 mice; LM: n_pre_ = 48 LFPs, 16 mice, n_post_ = 36 LFPs, 12 mice; AL: n_pre_ = 36 LFPs, 12 mice, n_post_ = 27 LFPs, 9 mice. (D) Power spectra of superficial layer LFPs within700 ms post-stimulus onset. Data are represented as mean ± 68% CI. V1: 4–8 Hz, p = 3.41 × 10^−3^; 8–12 Hz, p = 3.58 × 10^−4^; 12–30 Hz, p = 0.152; 30–50 Hz: p = 0.075; 50–80 Hz, p = 1.56 × 10^−4^. LM: 4–8 Hz, p = 0.031; 8–12 Hz, p = 0.091; 12–30 Hz, p = 0.681; 30–50 Hz, p = 0.084; 50–80 Hz, p = 0.015. AL: 4–8 Hz, p = 0.192; 8–12 Hz, p = 0.108; 12–30 Hz, p = 0.014; 30–50 Hz, p = 0.363; 50–80 Hz, p = 0.036, Mann-Whitney U test with FDR-BH correction. (E) The visual stimulus with SF and TF that induced the largest response in AL was used in the visual experience (SF = 0.03 cpd; TF = 6 Hz). The same stimulus was presented for 200 repeats per day for 4–6 days. Averaged LFP traces across mice before and after the visual experience were shown below. V1: n_pre_ = 60 LFPs, 20 mice; n_post_ = 51 LFPs, 17 mice. LM: n_pre_ = 48 LFPs, 16 mice; n_post_ = 30 LFPs, 10 mice. AL: n_pre_ = 36 LFPs, 12 mice; n_post_ = 33 LFPs, 11 mice. (F) Power spectra of superficial layer LFPs within 700 ms post-visual stimulus onset. Data are represented as mean ± 68% CI. V1: 4–8 Hz, p = 2.43 × 10^−5^; 8–12 Hz, p = 2.43 × 10^−5^; 12–30 Hz, p = 0.032; 30–50 Hz, p = 0.015; 50–80 Hz, p = 8.29 × 10^−4^. LM: 4–8 Hz, p = 2.23 × 10^−4^; 8–12 Hz, p = 0.012; 12–30 Hz, p = 0.039; 30–50 Hz, p = 0.906; 50–80 Hz, p = 0.510. AL: 4–8 Hz, p = 1.65 × 10^−3^; 8–12 Hz, p = 0.320; 12–30 Hz, p = 0.446; 30–50 Hz, p = 8.39 × 10^−3^; 50–80 Hz, p = 1.65 × 10^−3^, Mann-Whitney U test with FDR-BH correction. *p < 0.05, **p < 0.01, ***p < 0.001; n.s., p > 0.05.

To record simultaneous activity in V1 and HVAs, we used two silicon probes: one to record activity in V1 and the other to record activity in an HVA (either LM or AL) in head-fixed awake mice (Figure 1A). HVAs’ functional preferences for SFs and TFs were first characterized using pink noise stimuli that were spatially filtered around 5 SFs with the light intensity of each pixel changed in sinusoidal functions at 5 TFs (Figure 1A), which in turn formed 25 SF/TF combinations. To optimally target HVAs, retrograde or anterograde adeno-associated viruses (AAVs) that carried GFP or tdTomato were injected in V1 at 3 weeks prior to recordings to label projections between V1 and HVAs, thus labeling HVAs. Before recordings, the fluorescence in HVAs was checked under a stereoscope and was used to guide probe insertion together with stereotaxic coordinates. Representative histology showed GFP labeling V1 and LM (Figure 1B, top), tdTomato labeling AL/RL (rostrolateral) (Figure 1B, middle), and probe insertions in HVAs. SF and TF preferences of the visual areas were characterized using unit population-averaged responses (Figure S1). Within each mouse, unit firing rates were averaged across the population for each stimulus, then 25 responses were normalized by dividing by the largest response. Finally, for each region, the normalized responses were averaged across all mice and shown in heatmaps. The heatmaps revealed the stimuli that could maximally induce a response in LM (Figure S1B, SF = 0.12 cycles per degree [cpd], TF = 0.75 Hz), and in AL (Figure S1C, SF = 0.03 cpd, TF = 12 Hz), respectively.

### Persistent 4–8 Hz oscillations in V1 and HVA superficial layers after the visual experience

To induce LM or AL activity, the stimulus that maximally induced response in LM (stimulus A, SF = 0.12 cpd, TF = 0.75 Hz) was used in one set of experiments (Figure 1C), and the other stimulus that maximally induced response in AL (stimulus B, SF = 0.03 cpd, TF = 12 Hz) was used in the other set of experiments (Figure 1D). During the visual experience, mice were head-fixed in front of the visual stimulation monitor. The entrained stimulus (200 ms duration) was presented 200 times with a variable 5.5–7.5 s inter-stimulus interval between trials per day for 4 to 6 days. After the entrainment of SF and TF that maximally induced response in LM, the averaged local field potentials (LFPs) in V1 superficial, middle, and deep layers showed more prominent oscillatory activity (Figure 1C) in response to the entrained SF and TF. The superficial layer LFPs in LM and AL also showed oscillations with smaller amplitudes. The mean amplitudes of trough-to-peaks within 700 ms post-stimulus onset significantly increased in V1, in LM superficial layers, but not in AL (Figure S2). Power spectra of trial-averaged LFPs within 700 ms post-stimulus onset showed that 4–8 Hz power significantly increased in both V1 and LM superficial layer LFPs, but not in AL superficial layer LFPs (Figure 1D). LM and AL middle and deep layer LFPs did not show a significant change in 4–8 Hz power after the visual experience (FigureS3). To investigate whether the enhanced 4–8 Hz oscillation could be induced in AL, another set of mice was recorded after the entrainment of SF and TF that maximally induced response in AL (stimulus B visual experience). The averaged LFP traces in V1, LM, and AL showed more prominent oscillations in response to the entrained SF and TF (Figure 1E), and the 4–8 Hz power increased significantly in V1, and superficial layers in AL and LM (Figure 1F). These results demonstrated that visual familiarity could induce 4–8 Hz oscillations not only in V1 but also in superficial layers of LM and AL.

Because of the prevalence of the oscillations in the superficial layers and the existence of inter-areal feedforward and feedback projections in the superficial layers of V1,^35,36^ we then focused on analyzing superficial layer LFPs in V1, LM, and AL. To investigate whether the oscillation was specifically expressed in response to the entrained SF and TF, 4–8 Hz powers of superficial layer LFPs in response to stimuli at 25 combinations of SFs and TFs were calculated (Figure S4). 4–8 Hz powers of superficial layer LFPs significantly increased in responses to the entrained SF and TF, as well as to the SFs and TFs that were close to the entrained SF and TF after either stimulus A visual experience or stimulus B visual experience. This result demonstrated that the 4–8 Hz oscillations in superficial layers were most prominent in response to the entrained SF and TF, but also generalized in response to SFs and TFs that were close to the entrained SF and TF.

### 4–8 Hz phase synchronization was enhanced between V1 and LM, but not between V1 and AL, after the entrainment of stimulus that maximally induced response in LM

**Figure 2.**
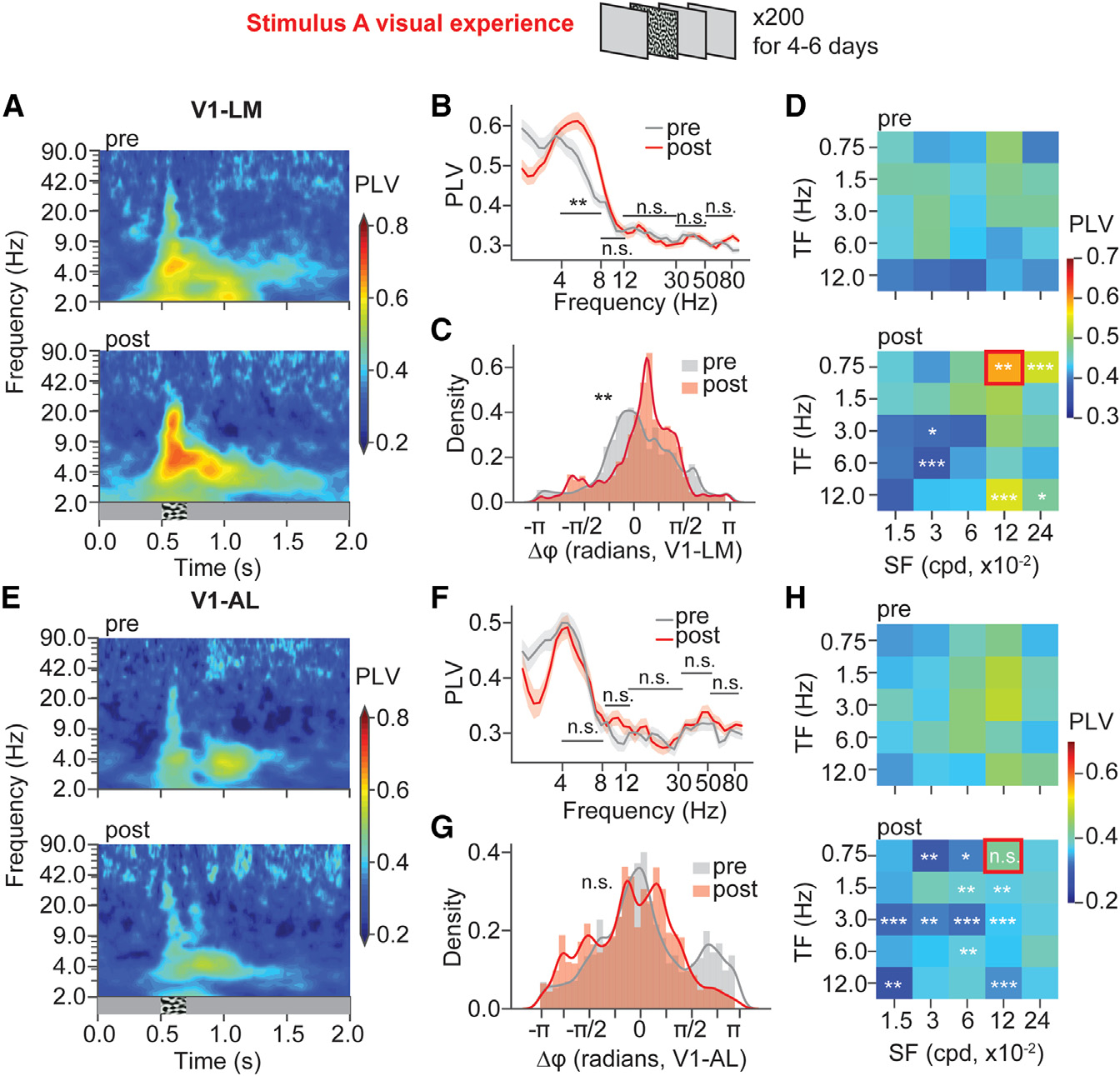
LM, but not AL, became more phase locked to V1 in 4–8 Hz after the entrainment of SF and TF that maximally induced response in LM (A) Phase-locking values (PLVs) of V1 and LM LFP pairs over time across frequencies were averaged and plotted in heatmaps. n_pre_ = 90 LFP pairs, 10 mice; n_post_ = 90 LFP pairs, 10 mice. (B) PLVs (median within 700 ms post-stimulus onset) of V1-LM LFP pairs across frequencies. Data are presented as mean ± 68% CI. 4–8 Hz, CLES = 0.648, p = 3.06 × 10^−3^; 8–12 Hz, CLES = 0.539, p = 0.611; 12–30 Hz, CLES = 0.506, p = 0.885; 30–50 Hz, CLES = 0.454, p = 0.611; 50–80 Hz, CLES = 0.492, p = 0.885, Mann-Whitney U test with FDR-BH correction. (C) 4–8 Hz oscillation phase differences between V1 and LM (within 700 ms post-stimulus onset) were plotted in density plots. D = 0.323, p = 5.02 × 10^−3^, Kuiper test. (D) Median 4–8 Hz PLVs of V1-LM LFP pairs in responses to five SFs and five TFs were plotted in heatmaps. Statistically significant differences between post-experience and pre-experience were labeled. See extended Table 3 in Data S1 for detailed statistics. (E) PLVs of V1 and AL LFP pairs over time across frequencies were averaged and plotted in heatmaps. n_pre_ = 81 LFP pairs, 9 mice; n_post_ = 54 LFP pairs, 6 mice. (F) PLVs (median within 700 ms post-stimulus onset) of V1-AL LFP pairs across frequencies. Data are presented as mean ± 68% CI. 4–8 Hz, CLES = 0.484, p = 0.758; 8–12 Hz, CLES = 0.606, p = 0.192; 12–30 Hz, CLES = 0.473, p = 0.745; 30–50 Hz, CLES = 0.528, p = 0.745; 50–80 Hz, CLES = 0.581, p = 0.277, Mann-Whitney U test with FDR-BH correction. (G) 4–8 Hz oscillation phase differences between V1 and AL (within 700 ms post-stimulus onset) were plotted in density plots. D = 0.198, p = 0.747, Kuiper test. (H) Median 4–8 Hz PLVs of V1-AL LFP pairs in responses to five SFs and five TFs were plotted in heatmaps. Statistically significant differences between post-experience and pre-experience for each stimulus were labeled. See extended Table 4 in Data S1 for detailed statistics. *p < 0.05, **p < 0.01, ***p < 0.001; n.s., p > 0.05.

After confirming that oscillations could be induced by visual familiarity in LM and AL, we next looked at whether the oscillations were synchronized inter-areally, which may serve for reliable inter-areal communication.^30,34,37^ To quantify the synchronization between V1 and HVAs, we calculated the phase-locking values (PLVs) between V1 superficial layer LFPs and LM superficial layer LFPs, or between V1 superficial layer LFPs and AL superficial layer LFPs. After the entrainment of SF and TF that maximally induced response in LM, V1 and LM exhibited enhanced 4–8 Hz phase-locking beyond the visual stimulus time window (Figure 2A), and the V1-LM PLV in 4–8 Hz averaged within 700 ms post-stimulus onset significantly increased after the visual experience (Figure 2B). Interestingly, V1 and AL did not show a significant change in 4–8 Hz phase-locking (Figures 2E and 2F). To infer the temporal relationship between V1 and HVAs, the 4–8 Hz phase differences between V1 and LM (Figure 2C) and between V1 and AL (Figure 2G) were plotted. Interestingly, the phase difference between V1 and LM (V1 phase – LM phase) showed a narrower and right-shifted distribution after the visual experience (Figure 2C), while the phase difference between V1 and AL did not show significant changes (Figure 2G). These results suggested that V1 became synchronized with LM, but not with AL, after the entrainment of SF and TF that could maximally induce a response in LM.

To examine whether the increased 4–8 Hz phase-locking between V1 and LM was specifically expressed in response to the entrained SF and TF, we built a heatmap with median 4–8 Hz PLVs (within 700 ms post-stimulus onset) for responses to the 25 stimuli. We then compared PLVs between post- and pre-visual experience for each stimulus. V1-LM PLVs increased in response to the entrained stimulus and stimuli with neighboring SFs and TFs. Meanwhile, the V1-LM PLVs significantly decreased in responses to stimuli that had distinct SFs or TFs from the entrained SF and TF (Figure 2D). On the contrary, there were no significant changes in PLVs between V1 and AL in response to the entrained SF and TF (Figure 2H), and intriguingly, the V1-AL PLVs were reduced in responses to the stimuli with SFs or TFs that were distinct from the entrained SF and TF (Figure 2H). This result demonstrated that 4–8 Hz phase synchronization between V1 and LM was expressed specifically in response to the entrained SF and TF.

### 4–8 Hz phase synchronization was enhanced between V1 and AL, but not between V1 and LM, after the entrainment of stimulus that maximally induced response in AL

**Figure 3.**
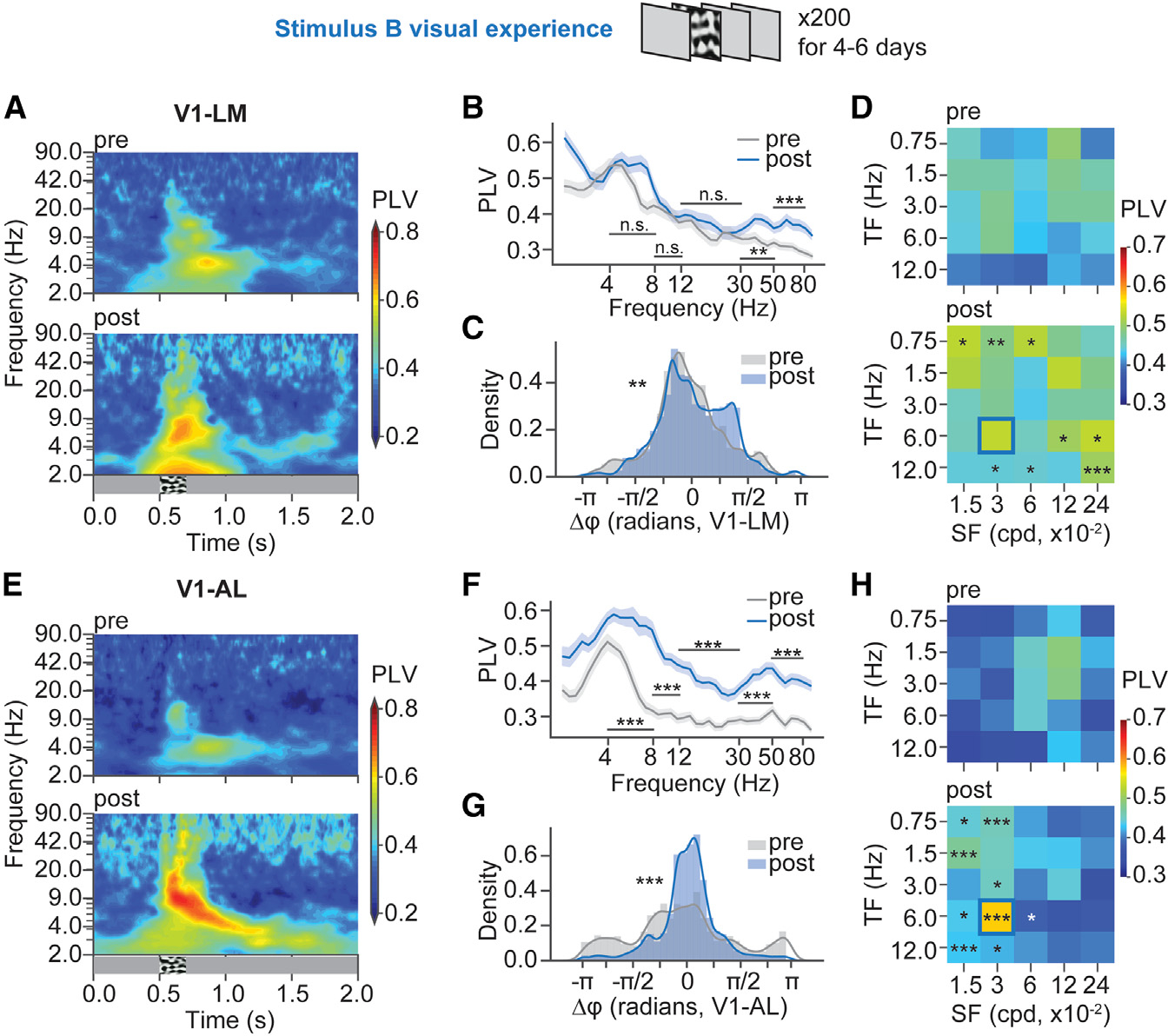
AL, but not LM, became more phase-locked to V1 in 4–8 Hz after the entrainment of SF and TF that maximally induced response in AL (A) PLVs of V1 and LM LFP pairs over time across frequencies were averaged and plotted in heatmaps. n_pre_ = 90 LFP pairs, 10 mice; n_post_ = 63 LFP pairs, 7 mice. (B) PLVs (median within 700 ms post-stimulus onset) of V1-LM LFP pairs across frequencies. Data are represented as mean ± 68% CI. 4–8 Hz, CLES = 0.523, p = 0.796; 8–12 Hz, CLES = 0.502, p = 0.972; 12–30 Hz, CLES = 0.597, p = 0.069; 30–50 Hz, CLES = 0.657, p = 2.50 × 10^−3^; 50–80 Hz, CLES = 0.790, p = 5.55 × 10^−9^, Mann-Whitney U test with FDR-BH correction. (C) 4–8 Hz oscillation phase differences between V1 and LM (within 700 ms post-stimulus onset) were plotted in density plots. D = 0.378, p = 4.68 × 10^−3^, Kuiper test. (D) Median 4–8 Hz PLVs of V1-LM LFP pairs in responses to five SFs and five TFs were plotted in heatmaps. Statistically significant differences between post experience and pre-experience for each visual stimulus were labeled. See extended Table 5 in Data S1 for detailed statistics. (E) PLVs of V1-AL LFP pairs over time across frequencies were averaged and plotted in heatmaps. n_pre_ = 81 LFP pairs, 9 mice; n_post_ = 54 LFP pairs, 6 mice. (F) PLVs (median within 700 ms post-stimulus onset) of V1-AL LFP pairs across frequencies. 4–8 Hz, CLES = 0.774, p = 2.49 × 10^−9^; 8–12 Hz, CLES = 0.844, p = 3.57 × 10^−13^; 12–30 Hz, CLES = 0.806, p = 3.63 × 10^−11^; 30–50 Hz, CLES = 0.806, p = 3.63 × 10^−11^; 50–80 Hz, CLES = 0.820, p = 8.84 × 10^−12^, Mann-Whitney U test with FDR-BH correction. (G) 4–8 Hz oscillation phase differences between V1 and AL (within 700 ms post-stimulus onset) were plotted in density plots. D = 0.454, p = 2.33 × 10^−4^, Kuiper test. (H) Median 4–8 Hz PLVs of V1-AL LFP pairs in responses to five SFs and five TFs were plotted in heatmaps. Statistically significant differences between post-experience and pre-experience for each stimulus were labeled. See extended Table 6 in Data S1 for detailed statistics. *p < 0.05, **p < 0.01, ***p < 0.001; n.s., p > 0.05.

In the other set of experiments where the entrained SF and TF could maximally induce a response in AL, V1, and LM showed no significant change in 4–8 Hz phase-locking (Figures 3A and 3B), while V1 and AL showed enhanced 4–8 Hz phase-locking persisting beyond the visual stimulation time window after the visual experience (Figures 3E and 3F). Interestingly, the 4–8 Hz phase difference between V1 and AL (Figure 3G), but not between V1 and LM (Figure 3C), became more concentrated after the stimulus B visual experience. This result demonstrated that V1 became phase synchronized with AL, but not with LM, after the entrainment of SF and TF that maximally induced response in AL. When we looked at the PLVs in responses to the 25 combinations of SFs and TFs, V1-LM PLVs did not show significant changes in response to the entrained SF and TF but showed significant increases in responses to other SFs and TFs (Figure 3D). On the contrary, V1-AL PLVs had a significant increase in response to the entrained SF and TF and the neighboring SFs and TFs (Figure 3H), demonstrating the specificity of the V1-AL 4–8 Hz phase synchronization to the entrained SF and TF.

These results demonstrated that V1 and HVA superficial layers were synchronized in 4–8 Hz after the visual experience, and the synchronization was selectively induced between V1 and the HVA that showed maximal response to the entrained SF and TF. Furthermore, the 4–8 Hz phase synchronization was specifically expressed in response to the entrained SF and TF, suggesting engagement of visual feature encoding neurons in the 4–8 Hz synchrony.

### Units spiked in clusters and became more phase-locked to local 4–8 Hz LFPs after the visual experience

**Figure 4.**
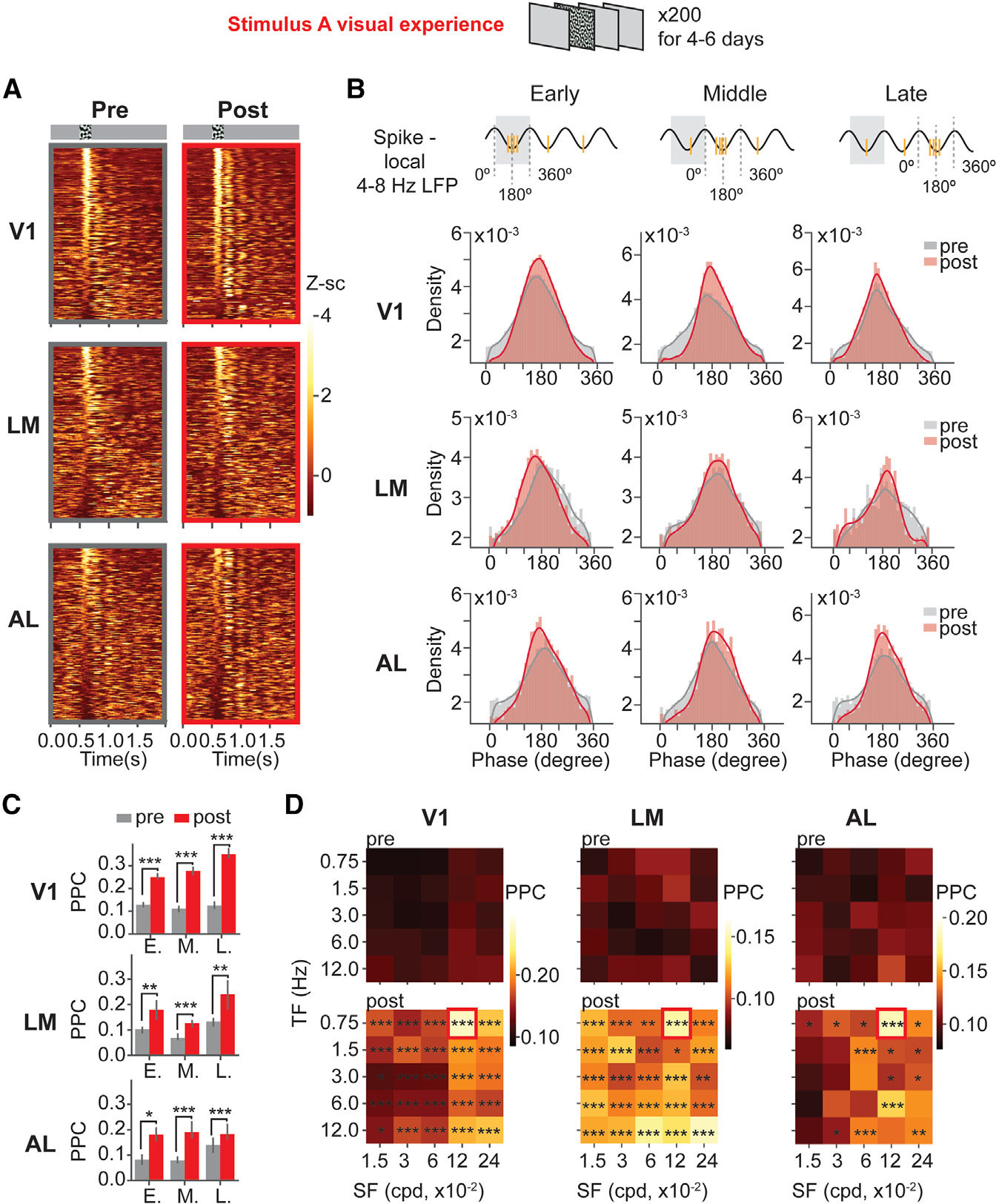
Single units in V1, LM, and AL spiked at more consistent 4–8 Hz phases after the entrainment of SF and TF that maximally induced response in LM (A) Units’ firing rate Z scores over time were plotted in heatmaps. V1: n_pre_, 1,171 units, 18 mice; n_post_, 1,617 units, 19 mice. LM: n_pre_, 965 units, 14 mice; n_post_, 1,173 units, 13 mice. AL: n_pre_, 783 mice, 12 mice; n_post_, 480 units, 6 mice. (B) Units were grouped into early-, middle-, and late-firing units based on the time windows of their peak firing rate Z score. 4–8 Hz spike phases of phase selective units in relation to local LFPs were plotted in density plots. (C) Pairwise phase consistency values (PPCs) (calculated using spike phases within 700 ms post-stimulus onset) of 4–8 Hz phase selective units are plotted in barplots. V1 Pre: n_early_, 497 units; n_middle_, 199 units; n_late_, 81 units, 18 mice. V1 Post: n_early_, 766 units; n_middle_, 320 units; n_late_, 147 units, 19 mice. LM Pre: n_early_, 158 units; n_middle_, 129 units; n_late_, 65 units, 14 mice. LM Post: n_early_, 117 units, n_middle_, 194 units; n_late_, 68 units, 13 mice. AL Pre: n_early_, 96 units; n_middle_, 141 units; n_late_, 87 units, 12 mice. AL Post: n_early_, 83 units; n_middle_, 92 units; n_late_, 62 units, 6 mice. Data are represented as median ± 68% CI. V1: early, p = 1.02 × 10^−28^; middle, p = 2.10 × 10^−20^; late, p = 4.14 × 10^−14^. LM: early, p = 2.51 × 10^−3^; middle, p = 1.2 × 10^−4^; late, p = 2.51 × 10^−3^. AL: early, p = 3.71 × 10^−6^; middle, p = 3.71 × 10^−6^; late, p = 0.015, Mann-Whitney U test with FDR-BH correction. (D) Averaged 4–8 Hz PPCs were plotted in heatmaps. The entrained stimulus is indicated by the red squares. The asterisks represent statistical significances in PPCs between pre- and post-visual experience. See extended Table 7 in Data S1 for detailed statistics. *p < 0.05, **p < 0.01, ***p < 0.001; n.s., p>0.05.

Next, we investigated how single-unit firing was modulated by local 4–8 Hz oscillations in V1, LM, and AL after the entrainment of SF and TF that maximally induced response in LM or AL. Unit population activity patterns were examined by plotting the Z score-normalized firing rates of all units in response to the entrained SF and TF. After the entrainment of SF and TF that maximally induced response in LM, V1 units spiked in clusters, and they grouped into stimulus-locked units and post-stimulus responsive units (Figure 4A). Units in LM or AL did not show clear spiking clusters as in V1. To examine whether the spiking clustering was modulated by local 4–8 Hz oscillations, we looked at the distributions of 4–8 Hz spike phases and calculated pairwise phase consistency (PPC) values to quantify unit phase-locking to 4–8 Hz local LFPs. Considering the units’ heterogeneous responses, we first separated units into early-firing (stimulus-locked), middle-firing, and late-firing (post-stimulus responsive) units based on their peak firing time windows (0.5–0.7, 0.7–0.9, and 0.9–1.2 s) (Figure 4B). After the entrainment of SF and TF that maximally induced response in LM, 4–8 Hz spike phases of early-firing (E.), middle-firing (M.), and late-firing (L.) units in V1 showed more concentrated distributions centered around 180° (4–8 Hz troughs). LM and AL 4–8 Hz spike phase distributions also became more concentrated, especially for the late-firing units. Then, we selected the phase-selective units that had non-uniform spike 4–8 Hz phase distributions (Rayleigh test p < 0.05) for PPC comparison. The visually locked (E.) and post-stimulus (M. and L.) phase-selective units in V1, LM, and AL all showed significantly increased PPCs after the visual experience (Figure 4C). This result demonstrated that unit spiking became more strongly modulated by local 4–8 Hz phases in V1, LM, and AL after the entrainment of the SF and TF that maximally induced response in LM.

We also examined whether the increased spike phase-locking was specific to the entrained SF and TF, by plotting median PPCs of phase-selective units in relation to SFs and TFs in heatmaps (Figure 4D). PPCs increased significantly in response to the entrained SF and TF, as well as to other SFs and TFs to a lesser extent (Figure 4D). This result demonstrated that units in V1, LM, and AL all became more phase-locked to local 4–8 Hz oscillations after the entrainment of SF and TF that maximally induced response in LM.

Similarly, in the other set of experiments where mice were entrained with the SF and TF that maximally induced response in AL, units spiked in clusters and were grouped into stimulus-locked units and post-stimulus responsive units after the visual experience (Figure S14A). The 4–8 Hz spike phases showed more concentrated distributions centered around 180°, most prominently in V1 (Figure S14B). When we quantified unit phase-locking using PPCs, both visually locked (E.) and post-stimulus (M. and L.) units in V1 showed significantly increased PPCs (Figure S14C, top). The stimulus-locked (E.) units in LM did not show significantly changed PPCs, but the post-stimulus middle-firing (M.) units in LM showed significantly increased PPCs after the visual experience (Figure S14C, middle). The visually locked (E.) units and the post-stimulus middle-firing (M.) units in AL showed significantly increased PPCs (Figure S14C, bottom). This result demonstrated that most units in V1, AL, and LM became more strongly modulated by 4–8 Hz phases after the entrainment of SF and TF that maximally induced response in AL.

Similar to the other set of experiments, PPCs increased most significantly in response to the entrained SF and TF, and also increased in response to other SFs and TFs to a lesser extent in V1, LM, and AL (Figure S14D). This result demonstrated that the spike 4–8 Hz phase-locking was most strongly expressed in response to the entrained SF and TF in V1, LM, and AL.

### Inter-areal functional connectivity changed after the visual experience

**Figure 5.**
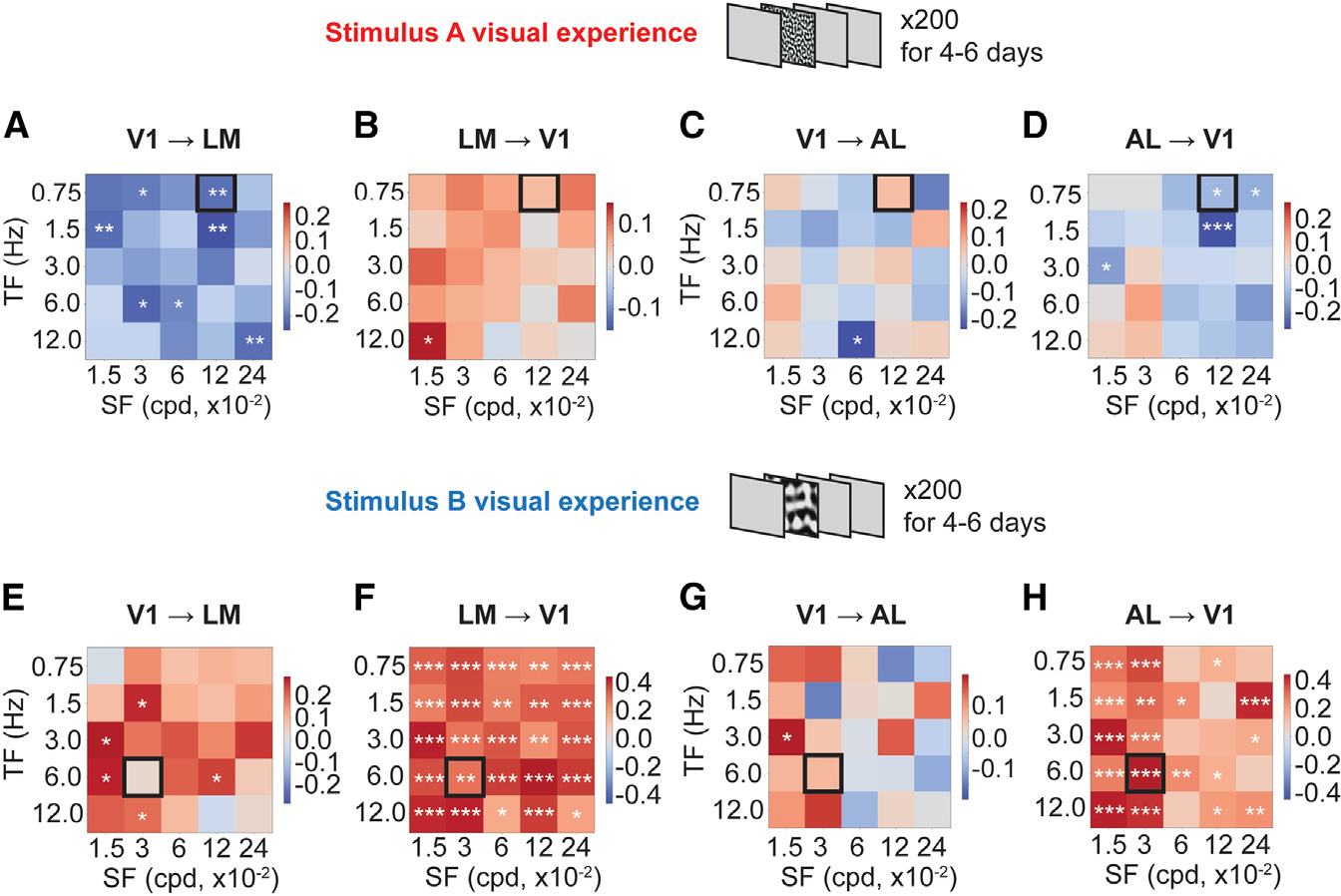
Functional connectivity between V1 and HVAs changed after the visual experience (A) Differences in V1 to LM functional connectivity between post-and pre-stimulus A visual experience were plotted in a heatmap. The entrained SF/TF combination is indicated by the black square. (B) Differences in LM to V1 functional connectivity between post-and pre-stimulus A visual experience were plotted in a heatmap. The entrained SF/TF combination is indicated by the black square. (C) Differences in V1 to AL functional connectivity between post- and pre-stimulus A visual experience were plotted in a heatmap. The entrained SF/TF combination is indicated by the black square. (D) Differences in AL to V1 functional connectivity between post-and pre-stimulus A visual experience were plotted in a heatmap. The entrained SF/TF combination is indicated by the black square. (E) Differences in V1 to LM functional connectivity between post-and pre-stimulus B visual experience were plotted in a heatmap. The entrained SF/TF combination is indicated by the black square. (F) Differences in LM to V1 functional connectivity between post- and pre-stimulus B visual experience were plotted in a heatmap. The entrained SF/TF combination is indicated by the black square. (G) Differences in V1 to AL functional connectivity between post- and pre-stimulus B visual experience were plotted in a heatmap. The entrained SF/TF combination is indicated by the black square. (H) Differences in AL to V1 functional connectivity between post- and pre-stimulus B visual experience were plotted in a heatmap. The entrained SF/TF combination is indicated by the black square. Monte Carlo simulations (10E–5 runs) were used to approximate the permutation test for each square in each difference matrix above. *p < 0.05, **p < 0.01, ***p < 0.001.

To infer whether there were changes in functional connectivity between V1 and HVAs after the visual experience, we performed directed information analysis using spike times within 1 s post-visual stimulus onset for each SF/TF combination.^26,38,39^ To minimize the influence of common inputs on computing the direct functional connectivity, a Markov order of 10 ms was used. To examine functional connectivity change after the visual experience, the post-experience functional connectivity was subtracted by the pre-experience functional connectivity for each SF/TF combination, and differences were plotted in heatmaps (Figure 5). After the entrainment of the SF/TF combination that maximally induced LM response, the V1 → LM feedforward functional connectivity was significantly reduced in response to the entrained SF/TF combination (Figure 5A) and was reduced to different extents in response to other SF/TF combinations. In contrast, the LM → V1 feedback functional connectivity showed a trend of increase in responses to multiple SF/TF combinations, despite statistical insignificance (Figure 5B). The V1 → AL feedforward functional connectivity showed variable changes in response to the SF/TF combinations (Figure 5C), while the AL → V1 feedback functional connectivity was significantly reduced in response to the entrained SF/TF combination (Figure 5D). This result suggested that the feedforward V1 → LM functional connectivity was reduced, and the feedback LM → V1 functional connectivity likely increased, while the feedback AL → V1 functional connectivity was reduced after the entrainment of SF and TF that maximally induced response in LM.

In the other set of experiments after the entrainment of the SF/TF combination that maximally induced response in AL, the V1 → LM feedforward functional connectivity showed a trend of increase in response to the entrained SF/TF combination (Figure 5E), and the LM → V1 feedback functional connectivity was significantly increased (Figure 5F) in response to the entrained SF/TF combination. Interestingly, the V1 → AL feedforward functional connectivity showed a trend of increase (Figure 5G) and the AL → V1 feedback functional connectivity was significantly increased in response to the entrained SF/TF combination (Figure 5H). The increased LM → V1 feedback functional connectivity was generalized (Figure 5F), while the increased AL → V1 feedback functional connectivity showed selectivity to the entrained SF to some extent (Figure 5H). This result suggested that feedforward V1 → LM and V1 → AL functional connectivity tended to increase, and the feedback LM → V1 and AL → V1 functional connectivity was significantly increased after the entrainment of the SF/TF combination that maximally induced response in AL.

### LM inactivation reduced V1 persistent oscillations after the visual experience

**Figure 6.**
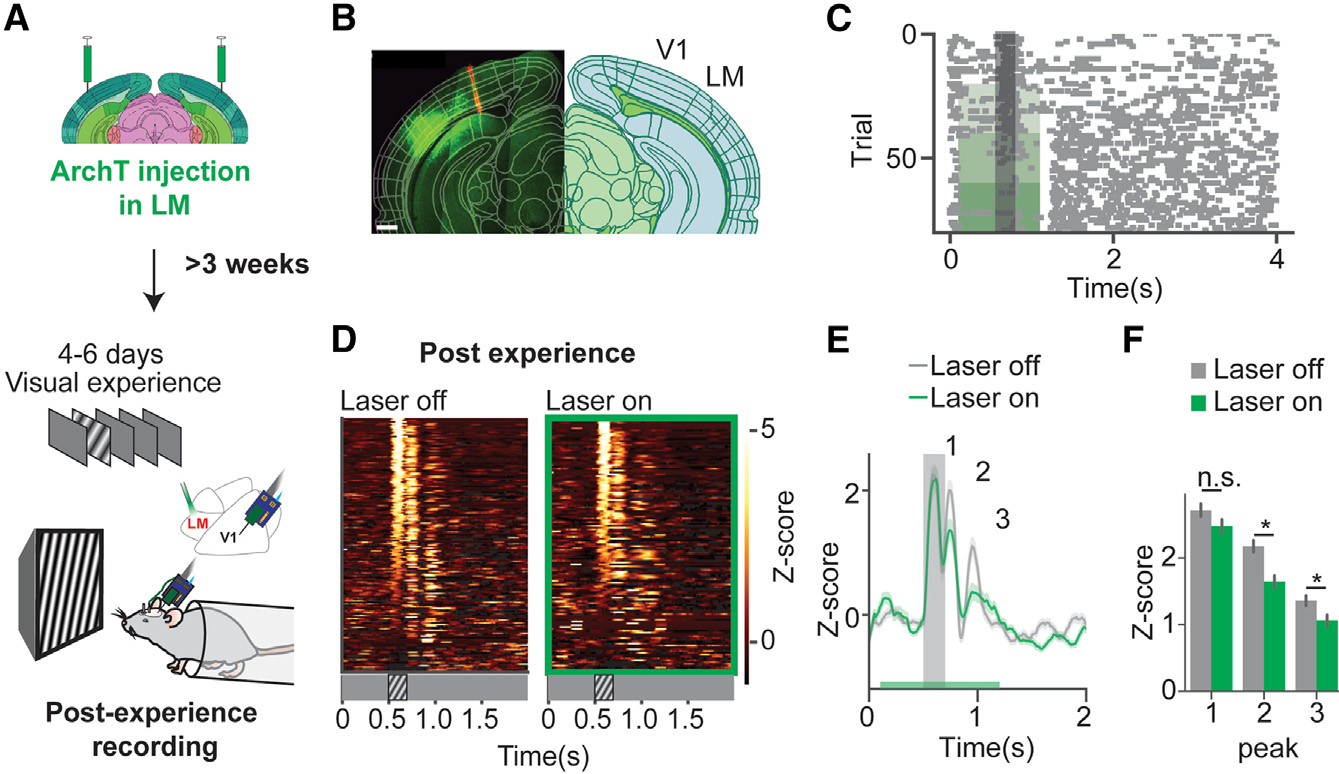
Optogenetic inhibition of LM reduced V1 post-stimulus oscillation amplitudes (A) AAV-ArchT-eYFP was injected in LM. Three weeks after virus injections, V1 activity was recorded, while LM was inactivated after the visual experience. The stimulus used in the visual experience was drifting gratings (SF = 0.04 cpd; TF = 2 Hz). (B) Histology (top) showed that ArchT-eYFP (green) was expressed outside V1, and probe track (red) was within V1. Scale bar, 500 μm. (C) The raster plot (bottom) showed a representative visually responsive LM unit that was inhibited when the 532 nm laser was turned on. The green shaded area represents the time window when the laser was on, and the color intensities represent laser powers (2, 4.5, and 10 mW). (D) Post-experience V1 units’ firing rate *Z* scores over time were plotted in heatmaps. (E) Averaged firing rate *Z* scores of all units after the visual experience were plotted (post: n_control_ = n_LM ArchT_ = 134 units, 5 mice). The oscillation peaks within three time windows (0.5–0.7, 0.7–0.9, and 0.9–1.1 s) are labeled with numbers. (F) Firing rate peak Z scores within the three time windows were plotted in a barplot. Data are represented as mean ± 68% CI. First peak, CLES = 0.468, p = 0.369; second peak, CLES = 0.410, p = 0.025; third peak, CLES = 0.415, p = 0.025, Mann-Whitney U test with FDR-BH correction. *p < 0.05, **p < 0.01, ***p < 0.001; n.s., not significant.

To test whether LM activity contributed to the persistent oscillatory activity in V1, we recorded V1 activity when LM activity was optogenetically inhibited by activating the AAV-CAG-ArchT virus locally in LM (Figure 6A). The ArchT expression was confirmed outside V1 and outside the V1 recording site (Figure 6B). A representative raster plot showed that a unit was inhibited when laser power increased (Figure 6C). To examine post-experience V1 oscillations when LM was inhibited, firing rate Z scores of the V1 unit population were plotted in heatmaps (Figure 6D). The population-averaged firing rate Z scores were significantly reduced at post-stimulus peaks but did not show significant changes in the visually locked peak (Figures 6E and 6F). The 4–8 Hz power of multi-unit activity was slightly lower when LM was inactivated (Figure S17). This result suggested that the experience-induced V1 post-stimulus responses were modulated by top-down activity from LM.

### LM inactivation following visual stimulus impaired visually cued “Go” behavior

**Figure 7.**
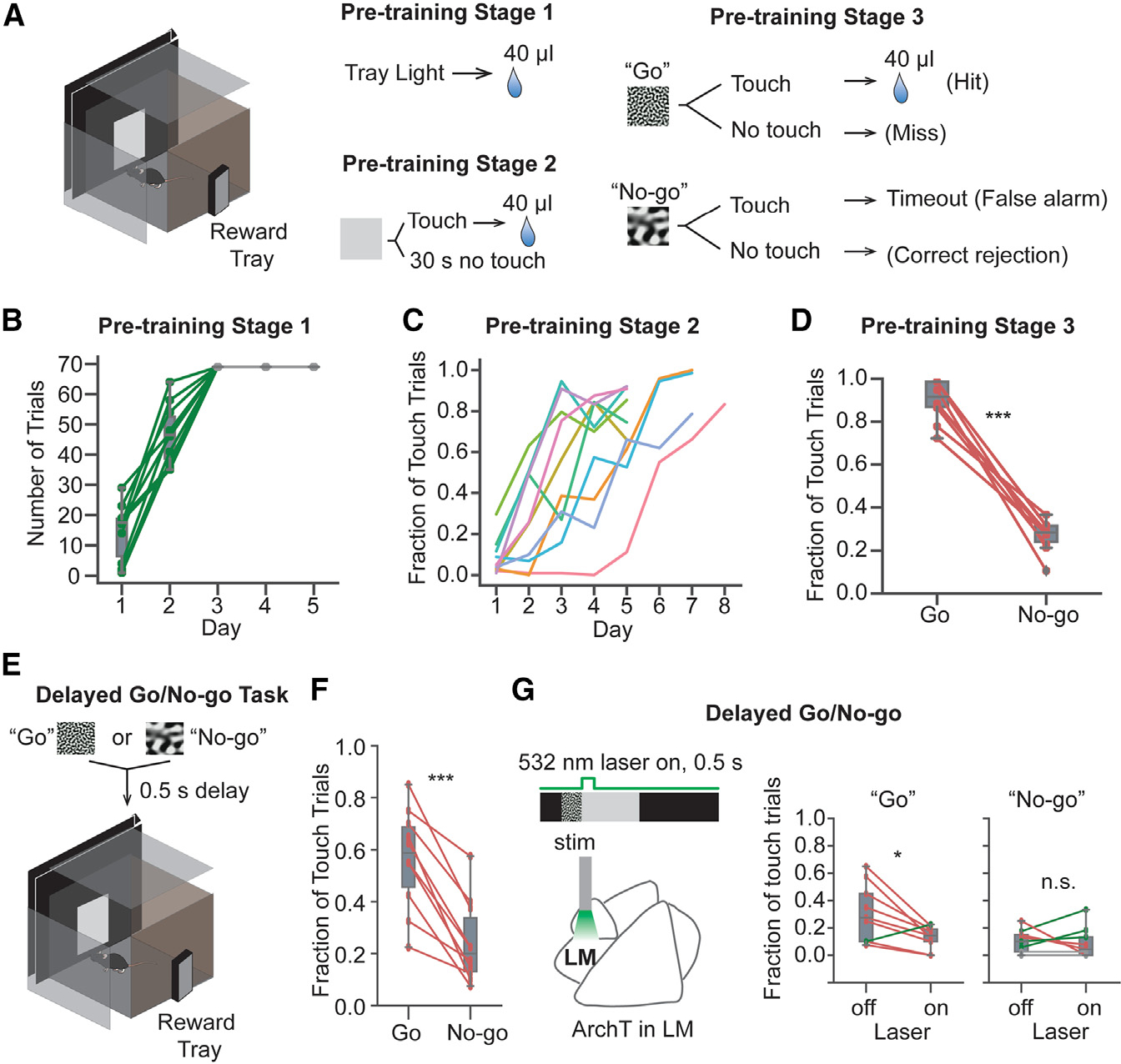
Optogenetic inactivation of LM post-stimulus activity impaired visually cued Go behavior (A) Mice were trained to perform a visually cued Go/No-go task using a touchscreen chamber through multiple pre-training stages. (B) Number of trials completed over days in pretraining stage 1. (C) Fraction of touch trials out of all trials over days in the pre-training stage 2. p = 1.9 × 10^−12^, n_Go_ = n_No-go_ = 9 mice, paired t test. (D) Fraction of touch trials on the best performance day in the pre-training stage 3. p = 7.27 × 10^−4^, n_Go_ = n_No-go_ = 9 mice, paired t test. (E) In the visually cued Go/No-go task, one visual stimulus was presented for 5 s at a time, followed by a gray screen displayed for 5.5 s. After the training, the mouse learned to touch the gray screen following the Go stimulus to get a sucrose water reward, and not to touch the gray screen following the No-go stimulus to avoid a time-out punishment. The Go stimulus is pink noise filtered at 0.12 cpd with light intensity change at 0.75 Hz. The No-go stimulus is pink noise filtered at 0.03 cpd with light intensity change at 0.75 Hz. (F) Fraction of touch trials on the best performance day in the visually cued Go/No-go task. (G) After the mice learned the task or their performance hit plateau, bilateral LM areas were inactivated by activating locally expressed ArchT using a 532 nm light through optic cannula on top of LM. The laser was turned on for 0.5 s following the visual stimulus before the touch response time window. The fractions of touch trials with laser turned off and turned on were plotted. Go trials, p = 0.019, n = 9 mice; No-go trials, p = 1, n = 9 mice. Wilcoxon signed-rank test. *p < 0.05, ** p < 0.01, ***p < 0.001; n.s., not significant.

It was reported that V1 persistent activity emerged following learning visually cued reward timing,^19^ and our results shown here suggest that V1 post-stimulus activity could be modulated by activity in higher-order visual areas. To test whether the top-down modulation from LM could affect visually cued delayed decision-making, we used a visually cued “Go/No-go” task using a touchscreen chamber with optogenetic inactivation of LM post-stimulus. Before the behavioral training, we injected AAV-CAG-ArchT locally in LM and implanted an optic cannula on top of the LM surface to enable inactivating LM later in the task. After the mice recovered from the surgery and had sufficient time to achieve viral expression, they were trained to perform the behavior task through several pre-training stages (Figure 7A). In pre-training stage 1, all mice were able to identify the reward port location and completed all 70 free reward trials per daily session (Figure 7B). In pre-training stage 2, all mice learned to touch the gray screen to receive sucrose water rewards (Figure 7C). In pre-training stage 3, a “Go” stimulus (pink noise with SF = 0.12 cpd, TF = 0.75 Hz), assuming the viewing distance is from the midpoint of the chamber to the screen, displayed for 5 s, or a No-go stimulus (pink noise with SF = 0.03 cpd, TF = 0.75 Hz), displayed for 5 s, was presented to mice. All mice touched the Go stimulus more than the No-go stimulus to receive rewards and avoid time-out punishment (Figure 7D). To test if mice could remember the familiar Go and No-go stimuli during the post-stimulus delay, in the training stage the Go stimulus or No-go stimulus was presented for 5 s, followed by a 5.5 s ray screen (Figure 7E). All animals touched the gray screen following the Go stimulus more than the gray screen following the No-go stimulus (Figure 7F). In the final stage, LM was inactivated by 532 nm light through the optic fiber for 0.5 s following the Go or the No-go stimulus (Figure 7G). The fractions of touch trials out of the first 40 trials in each condition were calculated and compared (Figure 7G). When LM was inactivated, the fraction of touch trials out of the Go trials was reduced compared with the fraction of touch trials when LM was not inactivated. Interestingly, some mice showed increased fractions of touch trials out of the No-go trials. In addition, we also showed that directly inactivating V1 post-stimulus activity impaired the visually cued Go behavior (Figure S20). Our results suggest that the visually cued Go behavior is modulated by LM post-stimulus activity.

## DISCUSSION

In this study, we demonstrated visual familiarity-induced persistent 4–8 Hz LFP oscillations in V1, LM, and AL, which were phase synchronized beyond the visual stimulation time window. This suggests stronger inter-areal communication between visual areas induced by familiarity and associated with visual recognition memory. LM was 4–8 Hz phase synchronized with V1 after the entrainment of SF and TF that maximally induced response in LM, while AL was 4–8 Hz phase synchronized with V1 after the entrainment of spatial and TF that maximally induced response in AL. Unit spikes in V1, LM, and AL were phase-locked and modulated by local 4–8 Hz phases after entrainment to the specific SF and TF. Optogenetic inactivation of LM during the delay period reduced V1 post-stimulus persistent oscillatory activity after the experience, suggesting the role of top-down modulation of V1 by HVAs in feature-specific visual familiarity. Finally, we discovered that inactivating LM impaired visually cued Go behavior, suggesting the necessity of post-stimulus LM activity in visually cued Go behavior.

In this study, we found that the persistent 4–8 Hz oscillations could be induced specifically in LM and AL in response to familiar visual stimuli with distinct features such as SF and TF. 4–8 Hz oscillations could be induced in LM but not in AL when the entrained SF and TF could maximally induce a response in LM. Similarly, familiarity-evoked oscillations could be induced in AL when the entrained SF and TF could maximally induce a response in AL. Similarly to the previously reported findings in V1, the oscillations in LM and AL were strongly expressed in response to the entrained SF and TF and generalized to other SFs and TFs that were close to the entrained ones.^22^ The enhanced post-experience oscillations in LM or AL were not attributed to baseline oscillation changes (Figures S5 and S6) or stimulus driving effect (Figure S9), and the oscillation power did not show a clear correlation with locomotion (Figures S7 and S8). Single-unit spiking was modulated by 4–8 Hz local phases in V1, LM, and AL after both types of visual experience. Visually locked and post-stimulus persistent units showed more concentrated distributions of spikes at 4–8 Hz troughs. Their 4–8 Hz phase-locking, indicated by the measure of PPC, significantly increased following the visual experience. The post-stimulus persistent spike clustering in LM or AL was less pronounced compared with the post-stimulus spike clustering in V1, which could be partially attributed to low firing rates in LM or AL (Figure S10). Nevertheless, the recorded units still showed increased 4–8 Hz phase locking, confirming that single-unit activity was greater modulated by 4–8 Hz oscillations in LM and AL after the visual experience. While synchronized firing may initially appear redundant, it is the synchronized excitability that enables efficient cross-regional communication and facilitates the synaptic plasticity crucial for learning and memory processes.^34,40^ Previous research has demonstrated that visual feature encoding engages various cortical areas beyond V1.^17,41^ Consequently, these oscillations may facilitate synchronized firing among feature-encoding neurons distributed across multiple cortical regions, potentially leading to the induction of synaptic plasticity between them.

Importantly, we discovered that these 4–8 Hz oscillations induced in V1 and HVAs by the familiar stimuli were synchronized with each other. LM became 4–8 Hz phase synchronized with V1 after the entrainment of SFs and TFs that maximally induced response in LM, while AL became 4–8 Hz phase synchronized with V1 after the entrainment of SFs and TFs that maximally induced response in AL. Synchronized oscillations have been proposed to mediate inter-areal communication through aligned excitable phases in two areas.^30,32,37^ The functional connections formed between neurons in V1 and HVAs preferentially responding to the entrained SF and TF became synchronized after the visual experience. The phase-locking between V1 and HVAs was greatest in response to the entrained SF and TF, suggesting that the synchronized neurons were likely the visual feature encoding neurons. Low-frequency oscillations were hypothesized to reflect feedback activities,^42,43^ and a recent study showed that a brief visual stimulus could induce 3–6 Hz activity propagating in a feedback direction to V1.^44^ We attempted to infer the directionality of the 4–8 Hz phase synchronization using the phase differences between regions, and calculated the phase slope indices to quantify the directionality, yet we did not find significant changes in 4–8 Hz LFP phase slope indices after the visual experience (Figure S15). The more concentrated 4–8 Hz phase differences after the visual experience could be alternatively explained by a common 4–8 Hz input from another brain region to V1 and higher visual areas, as studies suggested that a higher-order thalamic region exhibited theta^45^ and could change the gain of inter-cortical connections,^46–48^ possibly leading to inter-areal connectivity change.

To infer direct inter-areal functional connectivity using unit spikes, we performed directed information analyses using 10 ms Markov order to measure how activity in one area would predict the activity in the other area. Interestingly, the analyses revealed that LM activity tended to be more predictive of V1 activity after the entrainment of the SF/TF combination that maximally induced response in LM and was significantly more predictive of V1 activity after the entrainment of the SF/TF combination that maximally induced response in AL. AL activity was more predictive of V1 activity after the entrainment of the SF/TF combination that maximally induced response in AL, while it was less predictive of V1 activity after the entrainment of the SF/TF combination that maximally induced response in LM. The functional connectivity from LM to V1 tended to increase after either visual experience, which was likely partially attributed to the intermediate hierarchy of LM among visual cortical areas.^49^ Unlike the functional connectivity from LM to V1, the functional connectivity from AL to V1 was only increased after the entrainment of the SF/TF combination that maximally induced response in AL, suggesting more stimulus-specific increase.

We next showed that inactivating LM reduced post-stimulus persistent oscillation peaks in V1 after the visual experience, directly demonstrating the modulation of V1 persistent oscillations by LM. Interestingly, inactivating LM significantly reduced the response after the visual stimulation rather than during the visual stimulation after the visual experience, suggesting that the post-stimulus V1 activity might be under greater modulation of LM than the stimulus-locked V1 activity after the visual experience. Our findings are consistent with the recent study describing low-frequency feedback traveling waves evoked by a visual stimulus propagating from the association cortex along the cortical hierarchy back to V1.^44^ It was previously shown that activation of top-down projections from the cingulate cortex to the mouse V1 modulated inhibitory neuronal activity,^50^ which could dynamically control the oscillation frequency.^51^ The LM modulation of V1 oscillations could have a similar mechanism of regulation, especially in the post-stimulus delay time window. Interestingly, recent research demonstrated distinct population activity patterns during feedforward- and feedback-dominated activity patterns between V1 and V2 areas in primates. Specifically, V2 to V1 feedback population activity correlation was larger than the V1 to V2 feedforward population activity correlation after the visual stimulus offset but not during the visual stimulus.^52^ Our findings are consistent with this research and propose a distinct role for top-down modulation of V1 by HVAs during the post-stimulus delay period, which may be mediated by low-frequency persistent oscillations.

Given that visual familiarity could be encoded in the stimulus-locked response, what cognitive process could the persistent theta synchrony associate with? Previous research shows that theta phase-locking between V4 and the prefrontal cortex during the delay period of visual working memory was positively associated with performance in primates.^53^ Although theta dissociation between the visual cortex and the prefrontal cortex during visual stimulation was associated with sustained visual attention in mice.^54^ Simultaneous recordings in the cat V1 and parietal cortex showed that 4–12 Hz phase synchronization was enhanced when the cat saw a visual stimulus associated with a following Go behavior.^55,56^ Multiple mouse visual cortical areas other than V1 were associated with visually cued task performance. Inactivating LM or AL could impair contrast change detection or orientation discrimination,^16,17^ while inactivating PM or AM could impair visually cued subsequent behavior or visual working memory.^16,57^ In our visually cued Go/No-go behavioral experiment, we showed that inactivating a higher visual area LM during the delay after the visual stimulus impaired the visually cued Go behavior, providing evidence that persistent neuronal activity in LM was required for the visually cued Go behavior. Together with our data demonstrating top-down modulation of persistent oscillations in V1 by LM, these findings suggest that persistent theta oscillations may serve as a mechanism of feature-specific inter-areal synchronization between V1 and corresponding HVAs during recognition of a familiar stimulus. This hypothesis is consistent with the previous findings describing the involvement of HVAs in higher visual functions, including the perception of optical illusions.^9^ Future studies are necessary to test this hypothesis and reveal the role of the inter-areal synchronization mechanism in memory formation.

### Limitations of the study

A few limitations remain in the present study. First, the entrainment of visual stimulus that maximally induces response in AL also induced functional connectivity changes between V1 and LM as revealed by the directed information analysis, although the 4–8 Hz synchrony between V1 and LM did not show significant changes. It has been shown that simple visual feature processing could be distributed across multiple visual cortical areas.^17,41^ Consequently, the entrainment likely induces changes in HVAs other than AL. Second, to understand the effect of LM activity on familiarity-inducedoscillationsinV1,weinactivatedLMactivityusing ArchT that was locally expressed in LM. This strategy effectively inactivated LM activity but may also lead to the suppression of other frequency bands in addition to 4–8 Hz. Finally, the 4–8 Hz synchronybetween V1 and HV As was examined in a passive visual perception context. Thus, the role of 4–8 Hz oscillations in reward-related behavior needs to be studied in future experiments.

## STAR★METHODS

Detailed methods are provided in the online version of this paper and include the following:

### RESOURCE AVAILABILITY

#### Lead contact

Requests regarding resources, reagents, and any additional information should be directed to the lead contact, Alexander A Chubykin (Chubykin@purdue.edu). The address for correspondence is 915 Mitch Daniels Blvd., West Lafayette, IN 47907, Department of Biological Sciences, Purdue University.

#### Materials availability

No unique reagents were generated during this study.

#### Data and code availability

The analysis code for the directed information analysis is deposited at https://doi.org/10.5281/zenodo.10067600. The analysis code for the extracellular recordings and behavior studies is deposited at https://doi.org/10.5281/zenodo.10070360. Data and additional information are available from the corresponding author upon a reasonable request.

### EXPERIMENTAL MODEL AND SUBJECT PARTICIPANT DETAILS

Male and female C57BL/6 mice (Jackson Lab) at the age of 2–3 months were used for extracellular recording experiments and LM inactivation experiments. For the behavior experiment with V1 inactivation, male and female PV-Cre × Ai32 mice (Jackson Lab) at 2–3 months old were used. Mice were housed in a 12-h light/dark cycle with full access to water and chow food. All animal use was approved by Purdue IACUC and followed NIH guidelines.

### METHOD DETAILS

#### Headplate installation and virus injection

Before surgeries, mice were first anesthetized using 5% isoflurane in oxygen or room air (SomnoSuite system) in an induction chamber. After deep anesthesia was confirmed by a foot pinch, mice were transferred to a stereotaxic frame (Kopf or NeuroStar) and maintained anesthetized using 1.5–2% isoflurane delivered through a nose cone. A heating pad was put underneath the mouse’s body to prevent hypothermia. Eye ointment was applied to the mouse eyes to prevent dryness. The skin over the mouse skull was removed using sterile scissors, and 3% hydrogen peroxide was applied to the skull to remove connective tissues. V1, LM and AL were labeled with a permanent marker using stereotaxic coordinates (relative to lambda: V1: ±2.8 mm lateral, 0.5 mm anterior; LM: ±4.0 mm lateral, 1.0mm anterior; AL: ±3.8 mm lateral, 2.4 mm anterior). For some mice, retrograde or antegrade viruses that carry fluorescence (AAV1-CAG-tdTomato, rgAAV-CAG-GFP, Addgene #59462, #37825, gift from Edward Boyden) were injected into V1 to label HVAs (25–30 nL at 300 and 600 μm below the brain surface, Nanojet III). For LM optogenetic inhibition experiments, 50 nL of AAV5-CAG-ArchT-GFP (Addgene #29777 ^58^, gift from Edward Boyden) was locally injected in LM (±4.0 mm lateral, 1.0 mm anterior to the lambda) at 300 and 700 μm below the brain surface. A gold-plated reference pin (WPI 5482) was inserted through the skull and above the brain surface (0.5 mm anterior to the bregma), and a customized headplate was placed on top of the skull. The skull, the reference pin, and the headplate were covered with Metabond (Parkell S380) at the end. Carprofen (Rimadyl, 0.01 mL/g of 0.05 mg/mL for each mouse) and enrofloxacin (Baytril, 0.005 mL/g of 1 mg/mL for each mouse) were subcutaneously injected to the mice for three days post-surgery. Three weeks after the surgeries, mice started habituation to the head-fixation setup.

#### Visual experience paradigm and visual stimulation

To let the mice habituate to the setup, mice were head-fixed while allowed to freely run on a customized treadmill in a dark environment for at least 1.5 h per day for more than 3 days. A luminance linearly calibrated monitor was placed 20 cm in front of the mouse, showing a gray screen during the habituation. Visual stimuli were displayed during the passive visual experience and recording sessions. For the visual stimuli, a pink noise picture was first spatially filtered around five spatial frequencies (0.015, 0.03, 0.06, 0.12, 0.24 cycles/degree). For each filtered pink noise image, its light intensity at each pixel was changed in sinusoidal functions of time at five temporal frequencies (0.75, 1.5, 3, 6, 12 Hz), which, combined, generated 25 pink noise movies in total. The pink noise image (1920 ×1080 pixels) was first generated using MATLAB and then processed using Python. Visual stimuli were displayed using PsychoPy. For the passive visual experience, either one of two pink noise movies were used (0.12 cycles/degree and 0.75 Hz or 0.03 cycles/degree and 6 Hz). During the visual experience, one pink noise movie was presented for 0.2 s in each trial, for 200 trials per day for 4–6 days, with a variable 5.5–7.5 s Gy screen in between trials. During recording sessions, the stimulus used for the visual experience was presented repetitively for 20 trials, followed by pseudorandom presentations of 25 pink noise movies with 20 trials for each, and 5.5–7.5 s inter-trial intervals.

#### Extracellular recording preparation

After habituation or visual experience, extracellular activity in the visual cortex was recorded in head-fixed mice. Before the recording, fluorescence expressions in HVAs were examined under a stereoscope using a portable lamp (NIGHTSEA). Fluorescence was excited through a thinned skull or above the craniotomy over pre-labeled HVA areas and was examined using fluorophore compatible filters attached on the stereoscope. Two craniotomies were made over V1 and one HVA when the mouse was anesthetized using isoflurane on the stereotaxic frame. The mouse was then transferred to the head-fixation setup, and two 64 channel silicon probes^59^ were positioned above the two craniotomies using micromanipulators (NewScale). If bright fluorescence showed up around the prelabeled HVA location, the HVA probe was inserted at the brightest fluorescence location before the recording. In the case of dim fluorescence labeling, the HVA probe was inserted at the pre-labeled location. The V1 probe was inserted at the pre-labeled V1 location. Sterile saline was added on top of the brain surface before probe insertion. For some experiments, probes were dipped in fluorescent dye (DiD or DiO, Thermofisher, V22887, V22886) before insertion to label probe tracks. The probes were inserted at the speed of 50 μm/min to a depth of 950 μm. Ten minutes after probe insertions, data acquisition started.

For optogenetics experiments, the silicon probe recording preparation was similar to that described previously.^22,60^ In addition to silicon probe insertion, an optical fiber (Thorlabs, CFMLC12U-20 connected to FT200EMT, Ø200 μm Core, 0.39 NA) was placed above the brain surface. The optical fiber was connected to a 532 nm laser (OEM laser), which was triggered by a TTL signal 400 ms before the visual stimulation onset. Before experiments, the laser light power was measured at the tip of the optical fiber using a power meter (Thorlabs PM121D), and the power was kept between 4.5 and 10 mW for the optogenetics experiments.

#### Extracellular recording data acquisition and analysis

Data were acquired at 20 kHz using 64 channel silicon probes, Intan headstages, and OpenEphys^61^ acquisition system. Each trial recording was triggered using a TTL signal, and the visual stimulus onset timestamps were recorded by another TTL signal. Raw data was 300 Hz low-pass filtered and down-sampled to 1 kHz for LFP analysis. Raw data was also band-pass filtered between 300 and 6000 Hz for spike clustering using Kilosort/Kilosort2 using default clustering parameters. Clustered units were manually curated in Phy to remove noisy units. LFP and clustered spike data were analyzed using custom-written scripts in Python.

For LFP analysis, LFPs were first separated based on cortical depths. Within each recording, the LFP with the largest amplitude within 50–150 ms post-visual stimulation onset was identified as the middle layer LFP for each channel column. Then, the middle layer boundaries were identified as the depths 100 μm above and below the middle layer LFP channel. The LFP with the largest amplitude within 50–1000 ms post-visual stimulation onset within the channels above the middle layer upper boundary was then selected as the superficial layer LFP, and the LFP with the largest amplitude within 50–1000 ms post-visual stimulation onset within the channels below the middle layer lower boundary was selected as the deep layer LFP. LFP troughs and peaks were local extrema detected within 1000 ms post-stimulus onset, if their absolute amplitudes were larger than 25 μV and they were at least 50 ms apart. Trough-to-peak amplitude was the difference between adjacent trough and peak. Power spectra of LFPs were calculated using the Welch’s method using 700 ms Hanning windows, with 350 ms overlapping (SciPy).^62^ For power spectrum parameterization, each power spectrum (1–40 Hz) was fitted with a FOOOF model without a “knee”.^63^ Periodic peaks between 4 and 8 Hz were identified, and the adjusted periodic peak powers (adjusted by the aperiodic component) were reported as adjusted power. Exponents of aperiodic fits were reported to parameterize the aperiodic activity. For the inter-areal LFP phase-locking analysis, LFPs were filtered between 2 and 90 Hz using the Morlet wavelet method. LFP phases at each frequency were acquired using Hilbert transformation, and the inter-areal phase differences were calculated for each trial, and the vector sum of the phase differences across 20 trials were calculated as the phase-locking value (PLV) for each LFP pair. PLV was calculated using $\left|\frac{1}{N}\sum_{n=1}^{N}e^{j\Delta \varphi_{n}\left(t\right)}\right|$, where N is the number of trials, and $\Delta \varphi_{n}(t)$ is the phase difference at time t.^53,64^ Phase slope index of each LFP pair filtered at 4–8 Hz using morlet wavelet (3–4 cycles) was calculated (mne, phase_slope_index) to suggest directionality between the LFP pairs.^65,66^

For single unit analysis, peristimulus time histograms (PSTHs) were calculated for each unit by smoothing the spike histogram using a Gaussian kernel (with 30 ms as one standard deviation), and then the normalized response was calculated using the *Z* score of each unit’s PSTH ((FR_t_−mean(FR_t0−t1_))/std(FR_t0−t1_)). To identify preferred SF and TF of each visual cortical area, unit population responses to SFs and TFs were first normalized by the largest response within each mouse to make the largest response to 1. Then the normalized responses to 25 stimuli were averaged across mice to get the unit population normalized response heatmap for each area. To identify the hotspot of the heatmap for each visual cortical area, the original 5×5 map was first upsampled to 50×50 using bilinear interpolation and smoothed using a 2D Gaussian filter (sigma = 5 heatmap pixels). The pixel that had the highest value was selected as the hotspot for each region, and the corresponding SF and TF were considered as the preferred feature.^4^ Units were separated into early-, middle-, and late-firing units by ranking their time-averaged firing rates in three time windows: 0.5–0.7s, 0.7–0.9s, 0.9–1.2s. For intra-areal spike-phase analysis, the LFP from the channel where the unit showed the largest spike amplitude was chosen for each unit. The 4–8 Hz phases of the Butterworth bandpass filtered LFP at spike times of each trial were used for further spike-phase consistency analysis. To quantify spike phase consistency of each unit, a bias-free measure of pairwise-phase consistency (PPC) for each unit was used.^67^ PPC was calculated using $\frac{2}{N*(N-1)}*\sum_{n\;=\;1}^{N-1}\;\sum_{m\;=\;n+1}^{N}\;cos\left(d_{\left(\varphi_{m},\varphi_{n}\right)}\right)$, where N was the number of spikes of a unit, and $d_{\left(\varphi_{m},\varphi_{n}\right)}$ was the angular distance between phases of spike m and spike n.

Statistical tests were performed using statistical packages from SciPy, Pingouin, and Astropy. Data normality was tested using Shapiro-Wilk test. For normally distributed data, student’s t-tests were used. For non-normally distributed data, Mann–Whitney U tests were used. p values of multiple comparisons were corrected by keeping the false discovery rate at 5% using the Benjamini & Hochberg (FDR-BH) method. For comparisons between circular data distributions, Kuiper test was used.

#### Directed information analysis

We performed directed information analysis^26,38,39^ using spike times within 1 s post-visual stimulus onset for each SF/TF combination to assess differences in functional connectivity between units in V1 with AL and V1 with LM. Like in,^26^ each unit was fit with parametric models and then area-level connection strength was assessed using aggregated directed information values from those unit-level models. Unlike,^26^ which used minimum description length^69^ as a regularization penalty to select putative post-synaptic units, we used the lasso penalty for regularization. We next discuss the methodology in more detail.

##### Spike train preprocessing

For each recording, we only used spikes that occurred between 500 ms (stimulus onset) and 1500 ms for each trial to capture the persistent oscillatory period. Like in,^26^ time was discretized into 1 ms bins, resulting in binary-valued spike trains for each unit. To mitigate data imbalance, periods of time with no spikes from Y or candidate pre-synaptic units, and for which there were no spikes in the 100 ms prior, were not included.

##### Regression: Overview

To statistically assess the fit of sets of candidate pre-synaptic units for each unit Y, we modeled Y’s activity with a generalized linear model using the logit link function (logistic regression). Thus, for a given unit Y, a set of candidate pre-synaptic units, and time t, the likelihood of the binary variable Y(t) given the column vector of exogenous variables x (with a constant offset) and row vector of parameters θ was modeled as

(Equation 1) $$P(Y(t)=1|x;\theta )=\frac{1}{1+e^{-\theta x}}$$

Regularizers for parametric models assist in comparing models with different numbers of parameters and mitigate over-fitting. They balance low model error (low negative log likelihood) and using few parameters (low model complexity). In,^26^ there was an exhaustive search over many sets of candidate pre-synaptic units up to a cardinality of four. A set of candidate pre-synaptic units were then selected with a regularization penalty derived from the minimum description length principle.^69^ That process was computationally prohibitive for large sets of candidate pre-synaptic units. Instead, we used lasso-penalized logistic regression, resulting in an objective formula of the form.

$$-\frac{1}{T}\sum_{t=1}^{T}\;{log}_{2}P_{\theta }(Y(t)|Y(t-1),X(t-1))+\lambda |\theta {|}_{1}$$

where the first term is the empirical average negative log likelihood, $|\theta {|}_{1}$ sums up the magnitudes of the parameters, and λ is a hyperparameter. The hyper-parameter λ is chosen using cross-validation; that process is described below.

##### Regression: Pre-processing

The activity Y(t) was regressed using exogenous variables corresponding to prior activity from Y’s own past and the past of candidate pre-synaptic units. Two exogenous variables were constructed for Y’s own past, Y(t−1)+Y(t−2), specified to capture the refractory period, and Y(t−3)+…+Y(t−10) to capture self-dependence. For each candidate pre-synaptic unit X, one exogenous variable was used, X(t−1)+…+X(t−10). Lastly, a constant was used as an offset. As in,^26^ a Markov order of 10 ms was used to minimize the influence of common latent inputs on computing the direct functional connectivity.

##### Regression: Training/testing sets

For each unit Y, after an array of responses (Y(t) binary values) and exogeneous variables (comprised of Y’s past and the pasts of candidate pre-synaptic units) were collected, that set of data was split up 50/50 into training and testing sets. The split used stratification based on stimuli (SF/TF combinations). Each trial for each stimulus (SF/TF pair) was randomly partitioned in half, with data from half of the time-steps (i.e., rows of the data matrix) set as training data and the other half as testing data.

##### Regression: Cross-validation

The training set was used to select the hyper-parameter λ using 5-fold cross-validation and the final model based on the selected λ value. Within the cross-validation routine, the training set was split up into five parts and the following routine was repeated five times for each candidate hyper-parameter λ value. One part of the training set was held out (the ‘validation set’ for that iteration) while the model was fit using data from the remaining four parts (i.e., to select the parameter vector θ). The empirical negative log loss of the validation set data using the fitted model was evaluated. After that cross-validation routine completed, the empirical negative log loss from each fold was averaged, and the hyper-parameter λ value corresponding to the lowest empirical negative log loss was selected. All training data was then used to fit a new, final model (i.e., a new parameter vector θ) with that selected hyper-parameter λ value. Directed information was later calculated using testing data with that final model, with more details described below.

##### Regression: Implementation

Regularized regression was performed in Python (v3.8.8) using the LogisticRegressionCV function in the scikit-learn (v1.1.1) package with an ‘l1’ penalty, 5-folds, and with fixed hyper-parameter λ values. Twenty values between 10^−2^ and 10^1^ uniformly spaced on a log-scale were used as candidate hyper-parameter λ values. That range was selected to balance computation (fewer hyper-parameter λ values meant less computation) and modeling accuracy (a finer grid to search over will result in a model that better balances model complexity and data fit).

##### Directed information calculation: Overview

As in,^26^ directed information^70^ a non-parametric generalization of Granger causality,^71^ was used to measure the strength of unit level putative pre-synaptic connections. For a time-series Y(t) and a (possibly vector-valued) time-series X(t), the directed information from X to Y under a joint distribution P(X(1),…,X(T),Y(1),…,Y(T)) with a Markov-order one model that has a conditional distribution over Y parameterized by θ (and with a marginal distribution over Y parameterized by $\theta^{'}$) is

(Equation 2) $$I_{\theta }\left(X\to Y\right)=\frac{1}{T}\sum_{t=1}^{T}E_{P}\left[log_{2}\frac{P_{\theta }\left(Y\left(t\right)|Y\left(t\;-\;1\right),\;X\left(t\;-\;1\right)\right)}{P_{\theta^{\prime }}\left(Y\left(t\right)|Y\left(t\;-\;1\right)\right)}\right]$$

For each unit Y and set of candidate pre-synaptic units, we computed the empirical average of (Equation 2) with held-out testing dataset and the fitted θ and $\theta^{\prime }$. The empirical average is known to converge to the statistical average for stationary conditional distributions.^39^

##### Directed information: Area-wise assessment

For each unit Y, four models were fit using the regularized regression procedure described above, based on four sets of candidate pre-synaptic units as will be discussed shortly. In the following, we will describe the analysis for experiments where V1 and AL were simultaneously recorded. The analysis is the same for experiments where V1 and LM are simultaneously recorded.

One model, with parameter vector denoted as $\theta_{All}$, included all simultaneously recorded units (in V1 and AL) as candidate pre-synaptic units. A second model only included candidate pre-synaptic units from V1. A third model only included candidate pre-synaptic units from AL. The fourth model did not include any other units (only Y’s own past was used to construct exogenous variables for the regression). Denote the parameter vectors fitted for these four models as $\theta_{All}$, $\theta_{V1}$, $\theta_{AL}$, and $\theta_{None}$, respectively. Then directed information (Equation 2) for candidate pre-synaptic units from both V1 and AL was evaluated using testing data with $\theta_{All}$ for θ and $\theta_{None}$ as $\theta^{\prime }$ in (Equation 2). Denote that value as $I_{All}$. Directed information values $I_{V1}$ and $I_{AL}$ were likewise calculated for just candidate pre-synaptic units from V1 and from AL, respectively. Like in,^26^ the directed information values were normalized by the entropy of Y so that the normalized values could be interpreted as the proportion of uncertainty in modeling Y that was reduced by including the respective set of candidate pre-synaptic units.

We next attributed $I_{All}$ to V1 and AL. Unlike^26^ where the normalized directed information values were split up equally among putative pre-synaptic units (by construction limited to at most four total) and then combined based on unit locations, we used the Shapley value,^72^ which averages marginal contributions for different conditioning sets. For our purposes with attributing $I_{All}$ to two areas (V1 and AL), this simplifies to averaging the value of using units from each area (V1 and AL) conditioning on using units of the other area or not,

$$I_{V1}^{Shap\;}=\frac{1}{2}\left(I_{All}-I_{AL}\right)+\frac{1}{2}\left(I_{V1}-I_{None\;}\right)$$

$$I_{AL}^{Shap\;}=\frac{1}{2}\left(I_{AII}-I_{V1}\right)+\frac{1}{2}\left(I_{AL}-I_{None\;}\right).$$

We repeated the aforementioned evaluations for each unit Y in the experiments for which V1 and AL were simultaneously recorded. For each area (V1 and AL), we then calculated the median of attributed, normalized directed information values $I_{AL}^{Shap}$ and $I_{V1}^{Shap}$ over all post-synaptic units Y in that area for all identical experiments (same training status (pre-stimulus visual experience, post-stimulus A visual experience, post-stimulus B visual experience) and same SF/TF stimulus with V1 and AL simultaneously recorded).

To assess the overall differences due to stimulus A visual experience, we took differences between the median weights (directed information values) between pre-stimulus visual experience and post-stimulus A visual experience, for each ordered pair of areas (V1 and AL). We calculated similarly for stimulus B visual experience.

##### Significance testing

Like in,^26^ significance testing for the directed information analysis was performed using Monte Carlo approximations for two-sided permutation tests. 5E+5 iterations were used for each cell in the heatmaps in Figure 5. For each iteration, each weight used to calculate the median values from a pre-synaptic area to a unit in a post-synaptic area was relabeled as pre-stimulus visual experience or a post-stimulus (A or B) visual experience connection using corresponding proportions of connections in the pre- and post-stimulus visual experience experiments. For each iteration, the medians of shuffled edge strengths were calculated, and the difference of the median computed.

The Monte Carlo approximations were computed in Python(v3.8.8). We then corrected the p values by controlling the false discovery rate using the Benjamini-Hochberg procedure,^73^ implemented in the Python statsmodels(v0.13.2) package. These corrections were done separately for each pair of simultaneously recorded areas (V1/LM, V1/AL) and were done separately for stimulus A and stimulus B visual perceptual experiences.

#### Virus injection and fiber optic cannula implantation for behavior experiments

AAV5-CAG-ArchT-GFP (Addgene #29777^58^) was injected into LM (relative to lambda: ±4.0 mm lateral, 1.0 mm anterior, 25–30 nL each at 600 μm and 300 μm below the brain surface) in mice who were 1.5–2.5 months old. The detailed surgical procedure was described in the animal surgery method section. For the V1 inactivation experiment, virus injections were not necessary. Within 1–2 weeks after the virus injection, fiber optic cannula were implanted into the injection site bilaterally. Before the cannula implantation, the fiber protruding from the optic cannula was cut to 1–2 mm length, and the fiber tip was polished before implantation. For the implantation, the fiber optic cannula was placed on top of the craniotomy at the injection site, or above V1 (relative to lambda: ±3.0 mm lateral, 0.3 mm anterior), and then was stabilized by applying Metabond around the cannula. Post-operative care was the same as described earlier.

#### Behavior experiments

To train the mice to learn the task, mice were water-restricted (body weight kept >80% of the body weight on the day before water restriction) after they recovered from surgeries. Multiple pre-training stages were used similarly in other studies before they got trained for the visually cued delayed Go/No-go task.^9,74^ At least three days after animals recovered from the cannula implantation surgery, water bottles were removed from their home cages, and mice started to get habituated in the touchscreen chambers (Lafayette Instrument) and received water compensation in the reward port to learn where the reward was (“Free reward” stage). Before the mice got habituated in the touchscreen chambers, their body weights were recorded as their initial body weights. In the “Free reward” stage, the reward tray light was turned on to initiate a trial. After the mouse entered the reward port, a 40 μL water reward was delivered. The next trial initiated at 5 s after the mouse exited the reward port. Once the mouse could complete more than 70 trials per day for two consecutive days and they reached 85% of initial body weight, they were moved to the next stage, “Must touch” stage. In the “Must touch” stage, the mice must touch a gray screen to receive a sucrose water reward (1/12 volume/volume). Once they could complete at least 60% of trials per day for two consecutive days, they were moved to the “Go/No-go” stage. In the “Go/No-go” stage, one of the two pink noise stimuli was presented (5 s for LM inactivation experiments, 3 s for V1 inactivation experiments) at a time, and the mice learned to touch the “Go” stimulus to receive a reward, but not to touch the “No-go” stimulus to avoid a 10 s time-out punishment in addition to a 5 s inter-trial interval. Once the mice could touch more than 60% of the “Go” trials, and less than 40% of the “No-go” trials, they were moved to the “Delayed Go/No-go” stage, the visually cued delayed “Go/No-go” task. In the “Delayed Go/No-go” stage, a gray screen was presented following the pink noise movie, and the mice needed to touch the gray screen following the “Go” stimulus but not the gray screen following the “No-go” stimulus. The gray screen was presented for 5.5 s and the time window allowed for touch is 0.5 s after the gray screen onset. Once they could touch more than 60% of the delayed “Go” stimulus, and less than 40% of delayed “No-go” trials for at least two consecutive days, optical fiber cable was attached to the cannula using a ferrule mating sleeve on the mice to let them habituate to the fiber while performing the task for at least two more days before the test. In the case where the mice’s performance dropped with fiber attached, mice whose percentages of touched delayed “Go” trials stayed within 10% change from their best performance were moved to the final test stage as well. On the optogenetic inactivation days, LM or V1 was inactivated for 0.5 s following the “Go” or the “No-go” stimulus offset in a subset of trials, using a 532 nm laser or 473 nm laser (OEM laser), respectively. The laser power was kept at 3–5 mW as measured at the tip of the fibers. The first optogenetic inactivation was applied after the mice made five correct choices to ensure that the mice attended to the task.

In each training stage, mice were trained for one 90-min session daily until they reached the criteria to move to the next training stage. In each training stage, the mice were allowed to have a maximum of 70 rewards per 90-min daily session. If they completed less than 40 rewarded trials, free reward trials were added after the training to complete 40 rewarded trials to make sure the body weight was kept above 80% of their initial body weight. To train the mice to make the correct choices in the training stages, correction trials were used if the mice did not make the correct choices. In the correction trial, the same trial as the previous trial was displayed until the mice correctly completed the correction trial. The correction trials were not used in the final analyses.

For data analysis, the first 20 (V1 inactivation) or 40 (LM inactivation) regular trials from each condition were used in the final analyses, and the percentages of regular trials during which mice touched the screen out of all regular trials were calculated.

#### Mouse perfusion and histology

For some mice, brain tissues were extracted to visualize probe tracks, similarly to described previously.^60^ Briefly, after extracellular recordings, mouse was anesthetized with ketamine and xylazine and the anesthesia status was checked by a firm foot pinch. Once no reflex observed after a foot pinch, an incision was made at the mouse’s abdomen, and the heart was exposed after removing the skin and muscle. Mouse tissue was first perfused with 1x PBS until the liver cleared, then perfused with 4% PFA in PBS to fix the tissue. Brain tissue was extracted and sliced (50–100 μm) using a vibratome (Leica VT1000 S), and brain slices were imaged using a confocal microscope (Zeiss LSM710). Mouse brain reference atlas was mapped to the brain slice using Allen CCFv3.

### QUANTIFICATION AND STATISTICAL ANALYSIS

The details of quantification and statistics are reported in figure legends. For electrophysiology and behavior experiments, statistical tests were performed using statistical packages including SciPy, Pingouin,^68^ and Astropy. Data normality was tested using Shapiro-Wilk test. For normally distributed data, student’s t-tests were used. For non-normally distributed data, Mann–Whitney U tests were used. p values of multiple comparisons were corrected by keeping the false discovery rate at 5% using the Benjamini & Hochberg (FDR-BH) method. For comparisons between circular data distributions, Kuiper test was used. For the directed information analysis, the detailed quantification and statistics is detailed in the method details section.

## Supplementary Material

1

## Figures and Tables

**KEY RESOURCES TABLE:** 

REAGENT or RESOURCE	SOURCE	IDENTIFIER

Bacterial and virus strains		

AAV1-CAG-tdTomato	Addgene	Edward Boyden, Addgene viral prep # 59462-AAV1, RRID:Addgene_59462
rgAAV-CAG-GFP	Addgene	Edward Boyden, Addgene viral prep # 37825-AAVrg, RRID:Addgene_37825
AAV5-CAG-ArchT-GFP	Addgene	Edward Boyden, RRID:Addgene_29777^1^

Experimental models: Organisms/strains		

PV-Cre mice	The Jackson Lab	#008069, RRID:IMSR_JAX:008069
Ai32 mice	The Jackson Lab	#012569, RRID:IMSR_JAX:012569
Wild type C57BL/6 mice	The Jackson Lab	C57BL/6

Chemicals		

DiO	ThermoFisher	V22886
DiD	ThermoFisher	V22887

Software and algorithms		

Python	Python	https://www.python.org/
MATLAB	Mathworks	https://www.mathworks.com
PsychoPy	PsychoPy	https://www.psychopy.org/
ABET II	Lafayette Instrument	https://lafayettelifesciences.com/
Kilosort	Kilosort	https://github.com/MouseLand/Kilosort
allenCCF	Allen Brain Institute	https://github.com/cortex-lab/allenCCF
Pingouin	pingouin	https://pingouin-stats.org/

Other		

Stereotaxic frames	Neurostar or Kopf	https://neurostar.de/; https://kopfinstruments.com/
Isoflurane vaporizer	Parkland Scientific or SomnoSuite system	Parkland Scientific or Kent Scientific
Metabond	Metabond	Parkell S380
Portable LED light head (blue, green)	NIGHTSEA	RB-GO, GR
Micromanipulator	NewScale or Scientifica	https://www.newscaletech.com/ https://www.scientifica.uk.com/
OEM laser (532nm, 473nm)	OEM laser	http://www.oemlasersystems.com/
Arduino Uno	Arduino	https://www.arduino.cc/
Optical fiber and cannula	Thorlabs	FT200EMT, CFMLC12U-20
OpenEphys acquisition board	OpenEphys	https://open-ephys.org
Intan 128 channel headstage	Intan	RHD 128ch
64 channel silicon probes	Masmanidis Lab, UCLA	64D
Vibratome	Leica	VT1000, Leica
Touchscreen Chamber	Lafayette Instrument	Model 80614A

## Highlights

- Visual familiarity-induced 4–8 Hz oscillations in higher-order visual cortical areas
- Visual feature-specific 4–8 Hz synchrony between LM and V1 or between AL and V1
- Inactivating LM reduced V1 oscillation amplitudes
- Visual discrimination was impaired when LM was inactivated after the visual stimulus
