# Supplementary material for: Visual experience induces 4–8 Hz synchrony between V1 and higher-order visual areas
Source: Cell Rep. Author manuscript; Available in PMC 2024 Jan 16. (PMC10790627; doi:10.1016/j.celrep.2023.113482)
Supplement: 1 [file NIHMS1954767-supplement-1.pdf]

Cell Reports, Volume *42*

Supplemental information

Visual experience induces 4–8 Hz synchrony between V1 and higher-order visual areas

Yu Tang, Catherine Gervais, Rylann Moffitt, Sanghamitra Nareddula, Michael Zimmermann, Yididiya Y. Nadew, Christopher J. Quinn, Violeta Saldarriaga, Paige Edens, and Alexander A. Chubykin

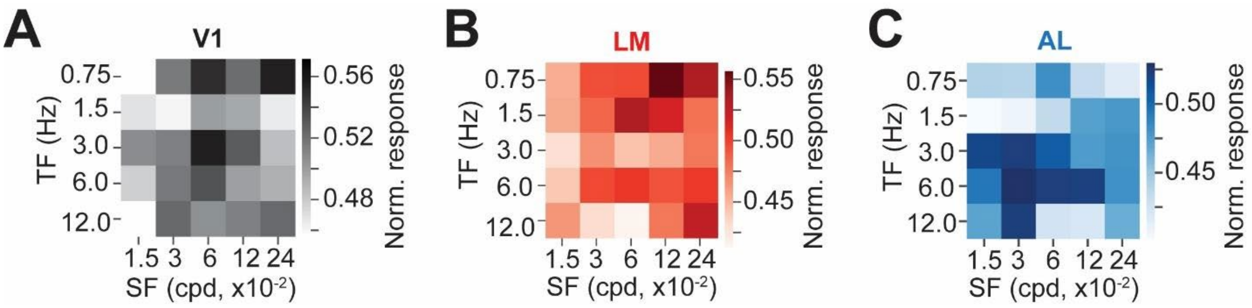

Figure S1. V1, LM, and AL units preferentially responded to subsets of SFs and TFs. Related to Figure 1.

(A) Averaged visually-locked responses of V1 units to twenty-five combinations of SFs and TFs were plotted in heatmaps. The visually-locked units' firing rates within each mouse were normalized (25 responses normalized to 0-1 range), and then averaged and plotted in heatmaps. n=18 mice.

(B) Averaged visually-locked responses of LM units to twenty-five combinations of SFs and TFs were plotted in heatmaps. The visually-locked units' firing rates within each mouse were normalized firing rates (25 responses normalized to 0-1 range). n=9 mice.

(C) Averaged visually-locked responses of AL units to twenty-five combinations of SFs and TFs were plotted in heatmaps. Visually-locked units' firing rates within each mouse were normalized firing rates (25 responses normalized to 0-1 range). n=6 mice.

*-p<0.05, **-p<0.01, ***-p<0.001, n.s.-p>0.05.

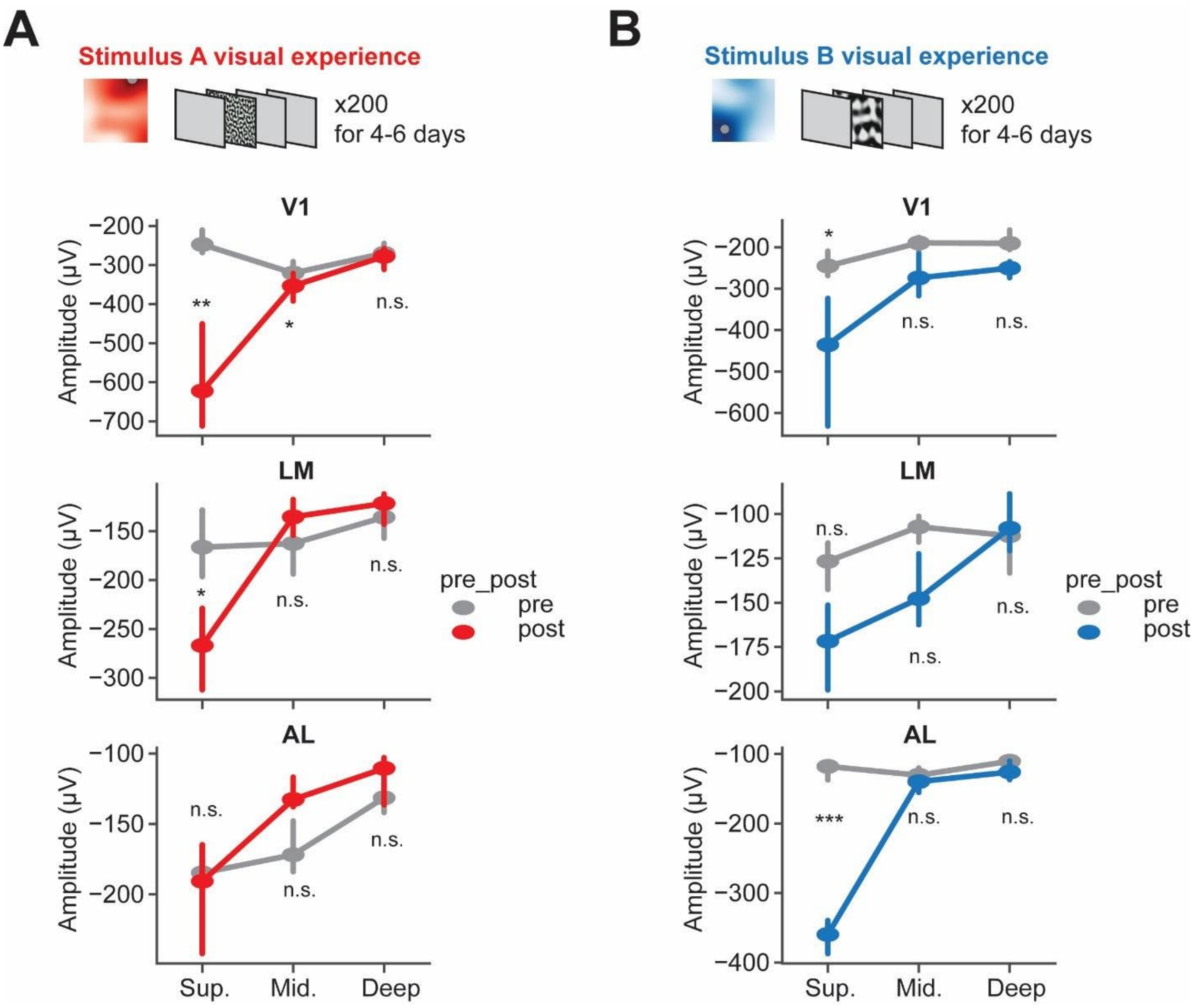

Figure S2. Trough-to-peak amplitudes increased in superficial layer LFPs after visual experience. Related to Figure 1.

(A) Trough-to-peak amplitudes (within 700 ms after the stimulus onset) before and after stimulus A visual experience were plotted. V1: Sup.: $p=1.11 \times 10^{-4}$, n_{pre}=60 LFPs, 20 mice, n_{post}= 60 LFPs, 20 mice, Mid.: p=0.035, n_{pre}=60 LFPs, 20 mice, n_{post}= 60 LFPs, 20 mice, Deep: p=0.408, n_{pre}=59 LFPs, 20 mice, n_{post}= 60 LFPs, 20 mice; LM: Sup.: p=0.029, n_{pre}=48 LFPs, 16 mice, n_{post}= 36 LFPs, 12 mice, Mid.: p=0.322, n_{pre}=48 LFPs, 16 mice, n_{post}= 36 LFPs, 12 mice, Deep: p=0.709, n_{pre}=47 LFPs, 16 mice, n_{post}= 36 LFPs, 12 mice; AL: Sup.: p=0.307, n_{pre}=36 LFPs, 12 mice, n_{post}= 27 LFPs, 9 mice, Mid.: p=0.301, n_{pre}=36 LFPs, 12 mice, n_{post}=

27 LFPs, 9 mice, Deep: p=0.622, n_{pre}=36 LFPs, 12 mice, n_{post}= 27 LFPs, 9 mice, Mann-Whitney U test with FDR-BH correction.

(B) Trough-to-peak amplitudes (within 700 ms after the stimulus onset) before and after stimulus B visual experience were plotted. V1 Sup.: p=0.025, n_{pre}=60 LFPs, 20 mice, n_{post}= 51 LFPs, 17 mice; V1 Mid.: p=0.240, n_{pre}=60 LFPs, 20 mice, n_{post}= 51 LFPs, 17 mice; V1 Deep: p=0.129, n_{pre}=60 LFPs, 20 mice, n_{post}= 51 LFPs, 17 mice; LM Sup.: p=0.055, n_{pre}=48 LFPs, 16 mice, n_{post}= 24 LFPs, 8 mice; LM Mid.: p=0.279, n_{pre}=48 LFPs, 16 mice, n_{post}= 22 LFPs, 8 mice; LM Deep: p=0.940, n_{pre}=47 LFPs, 16 mice, n_{post}= 23 LFPs, 8 mice; AL Sup.: $p=2.35\times10^{-5}$, n_{pre}=36 LFPs, 12 mice, n_{post}= 27 LFPs, 9 mice; AL Mid.: p=0.163, n_{pre}=36 LFPs, 12 mice, n_{post}= 27 LFPs, 9 mice; AL Deep: p=0.603, n_{pre}=36 LFPs, 12 mice, n_{post}= 27 LFPs, 9 mice, Mann-Whitney U test with FDR-BH correction.

*-p<0.05, **-p<0.01, ***-p<0.001, n.s.-p>0.05.

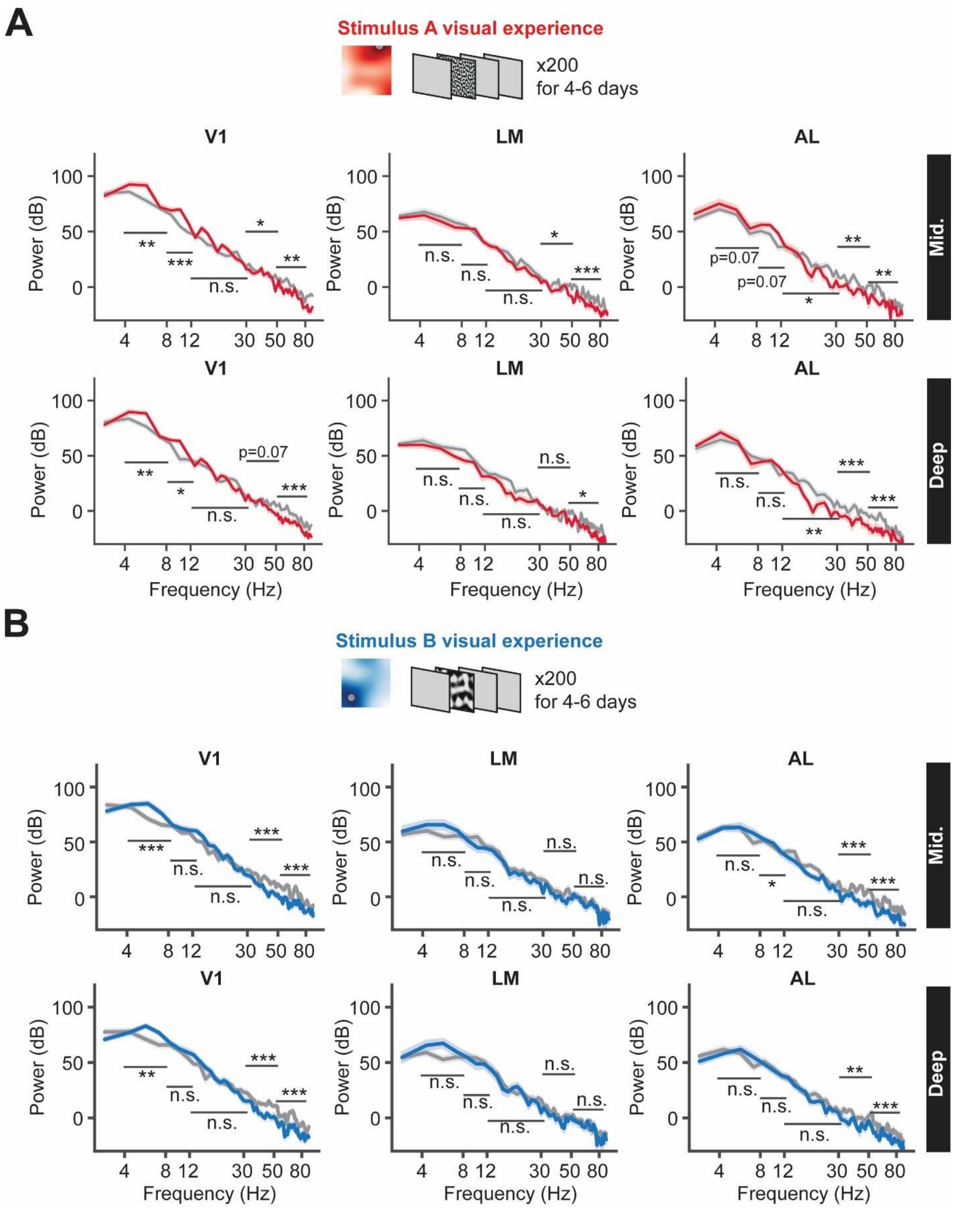
A
Stimulus A visual experience
x200
for 4-6 days
V1
LM
AL
Mid.
Deep
Power (dB)
Frequency (Hz)
n.s.
p=0.07
B
Stimulus B visual experience
x200
for 4-6 days

Figure S3. 4-8 Hz power of middle and deep layer LFPs increased in V1, but not in LM or AL. Related to Figure 1.

(A) Power spectra of middle layer (top) and deep layer (bottom) LFPs within 700 ms post-stimulus onset before and after stimulus A visual experience. Data were presented as mean ± 68% CI. V1 Mid.: 4-8 Hz: $p=2.22\times10^{-3}$, 8-12 Hz: $p=5.78\times10^{-4}$, 12-30 Hz: $p=0.956$, 30-50 Hz: $p=0.013$, 50-80 Hz: $p=2.21\times10^{-3}$; LM Mid.: 4-8 Hz: $p=0.524$, 8-12 Hz: $p=0.397$, 12-30 Hz: $p=0.089$, 30-50 Hz: $p=0.027$, 50-80 Hz: $p=2.83\times10^{-4}$; AL Mid.: 4-8 Hz: $p=0.074$, 8-12 Hz: $p=0.068$, 12-30 Hz: $p=0.014$, 30-50 Hz: $p=1.94\times10^{-3}$, 50-80 Hz: $p=3.95\times10^{-3}$; V1 Deep: 4-8 Hz: $p=6.51\times10^{-3}$, 8-12 Hz: $p=0.016$, 12-30 Hz: $p=0.373$, 30-50 Hz: $p=0.073$, 50-80 Hz: $p=2.94\times10^{-5}$; LM Deep: 4-8 Hz: $p=0.967$, 8-12 Hz: $p=0.669$, 12-30 Hz: $p=0.136$, 30-50 Hz: $p=0.669$, 50-80 Hz: $p=0.012$; AL Deep: 4-8 Hz: $p=0.287$, 8-12 Hz: $p=0.408$, 12-30 Hz: $p=5.04\times10^{-3}$, 30-50 Hz: $p=7.03\times10^{-4}$, 50-80 Hz: $p=7.03\times10^{-4}$, Mann-Whitney U test with FDR-BH correction.

(B) Power spectra of middle layer (top) and deep layer (bottom) LFPs within 700 ms post-stimulus onset before and after stimulus B visual experience. Data were presented as mean ± 68% CI. V1 Mid.: 4-8 Hz: $p=6.61\times10^{-5}$, 8-12 Hz: $p=0.487$, 12-30 Hz: $p=0.487$, 30-50 Hz: $p=1.08\times10^{-5}$, 50-80 Hz: $p=4.46\times10^{-6}$; LM Mid.: 4-8 Hz: $p=0.297$, 8-12 Hz: $p=0.337$, 12-30 Hz: $p=0.337$, 30-50 Hz: $p=0.337$, 50-80 Hz: $p=0.495$; AL Mid.: 4-8 Hz: $p=0.679$, 8-12 Hz: $p=0.679$, 12-30 Hz: $p=0.047$, 30-50 Hz: $p=8.30\times10^{-5}$, 50-80 Hz: $p=3.62\times10^{-5}$; V1 Deep: 4-8 Hz: $p=9.54\times10^{-3}$, 8-12 Hz: $p=0.309$, 12-30 Hz: $p=0.706$, 30-50 Hz: $p=4.71\times10^{-4}$, 50-80 Hz: $p=6.03\times10^{-7}$; LM Deep: 4-8 Hz: $p=0.229$, 8-12 Hz: $p=0.896$, 12-30 Hz: $p=0.896$, 30-50 Hz: $p=0.229$, 50-80 Hz: $p=0.896$; AL Deep: 4-8 Hz: $p=0.927$, 8-12 Hz: $p=0.458$, 12-30 Hz: $p=0.678$, 30-50 Hz: $p=9.15\times10^{-3}$, 50-80 Hz: $p=7.52\times10^{-4}$, Mann-Whitney U test with FDR-BH correction.

*-$p<0.05$, **-$p<0.01$, ***-$p<0.001$, n.s.-$p>0.05$.

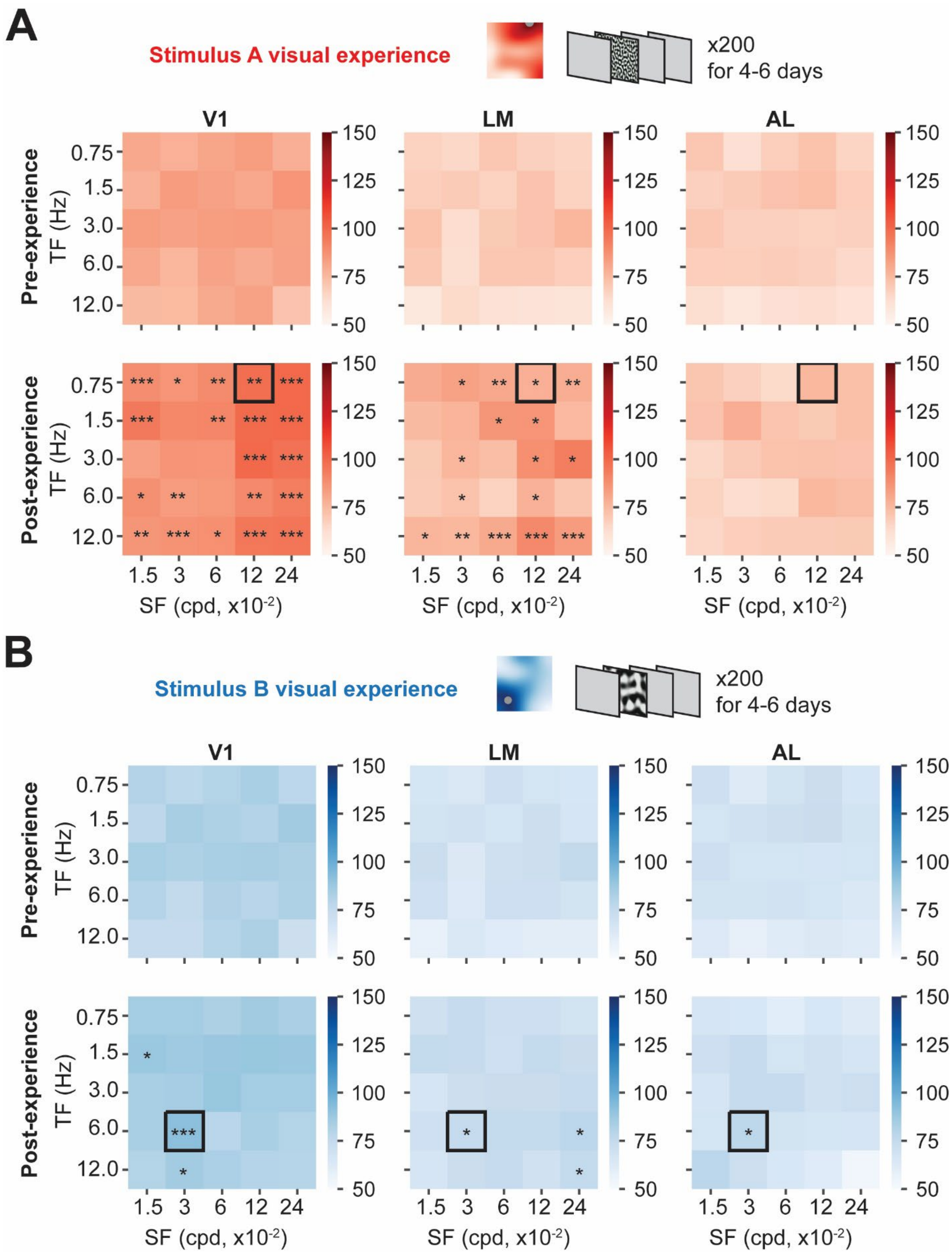
A
Stimulus A visual experience
x200
for 4-6 days
V1
LM
AL
Pre-experience
Post-experience
TF (Hz)
0.75
1.5
3.0
6.0
12.0
SF (cpd, x10-2)
1.5
3
6
12
24
150
125
100
75
50
B
Stimulus B visual experience
x200
for 4-6 days

Figure S4. 4-8 Hz power of superficial layer LFPs in response to twenty-five combinations of SFs and TFs. Related to Figure 1.

(A) Averaged 4-8 Hz powers of LFPs within 700 ms post-stimulus onset were plotted in heatmaps. The stimulus used in stimulus A visual experience was indicated by the black square. Statistically significant differences between post- and pre- experience for each stimulus were labeled. See extended table 1 for detailed statistics.

(B) Averaged 4-8 Hz powers of LFPs within 700 ms post-stimulus onset were plotted in heatmaps. The stimulus used in stimulus B visual experience was indicated by the black square. Statistically significant differences between post- and pre- experience for each stimulus were labeled. See extended table 2 for detailed statistics.

*-$p<0.05$, **-$p<0.01$, ***-$p<0.001$, n.s.-$p>0.05$.

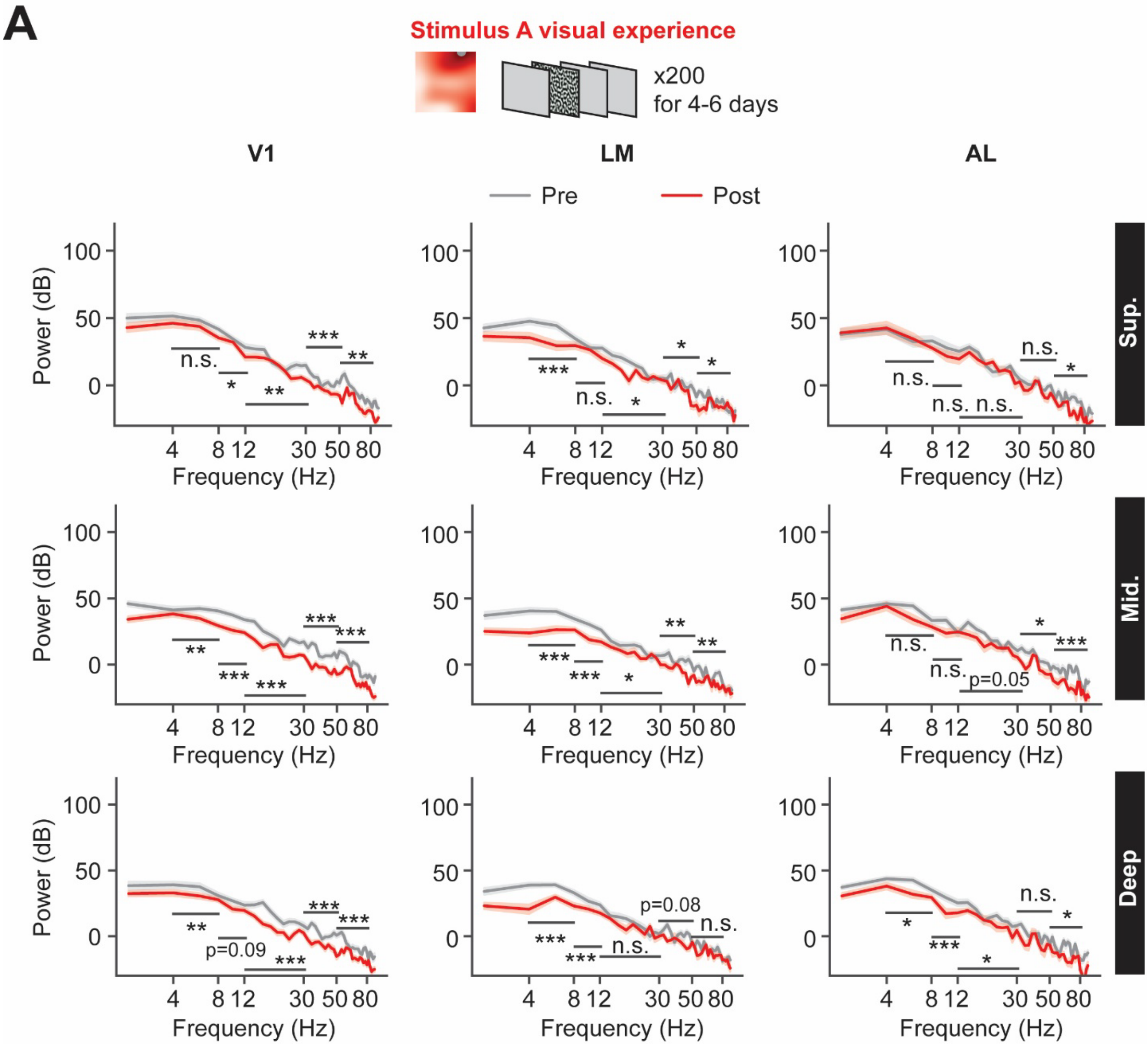

Figure S5. Pre-stimulus 4-8 Hz power did not increase in V1, LM, or AL, after the entrainment of SF and TF that maximally induced response in LM. Related to Figure 1.

(A) Power spectra of superficial, middle, and deep layer LFPs during the 500 ms before the stimulus onset, before and after the stimulus A visual experience. Data were presented as mean ± 68% CI. Mann-Whitney U test with FDR-BH correction. See extended table 17 for detailed statistics.

*-p<0.05, **-p<0.01, ***-p<0.001, n.s.-p>0.05.

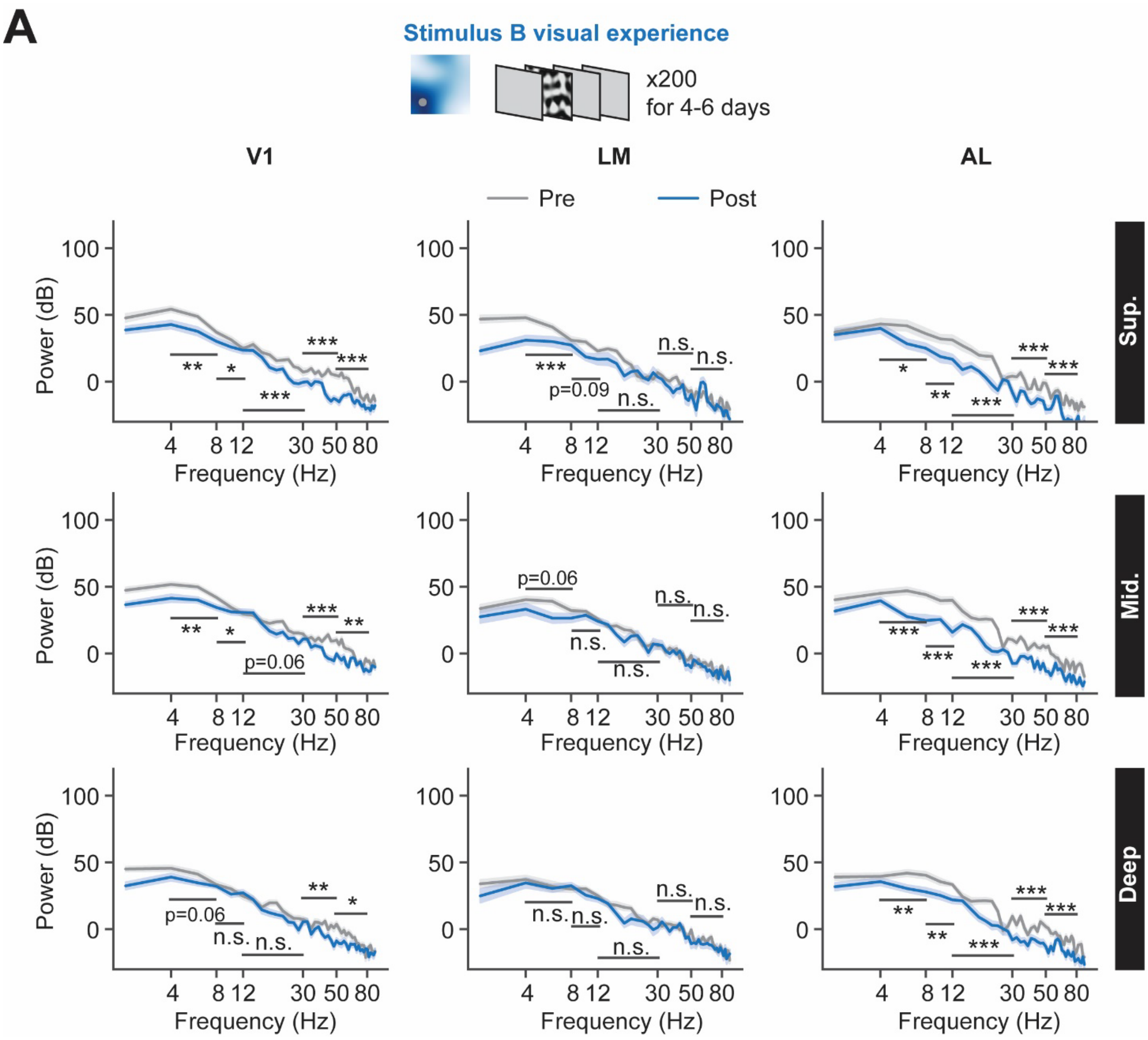

Figure S6. Pre-stimulus 4-8 Hz power did not increase in V1, LM, or AL, after the entrainment of SF and TF that maximally induced response in AL. Related to Figure 1.

(A) Power spectra of superficial, middle, and deep layer LFPs during the 500 ms before the stimulus onset, before and after the stimulus B visual experience. Data were presented as mean ± 68% CI. Mann-Whitney U test with FDR-BH correction. See extended table 18 for detailed statistics.

*-p<0.05, **-p<0.01, ***-p<0.001, n.s.-p>0.05.

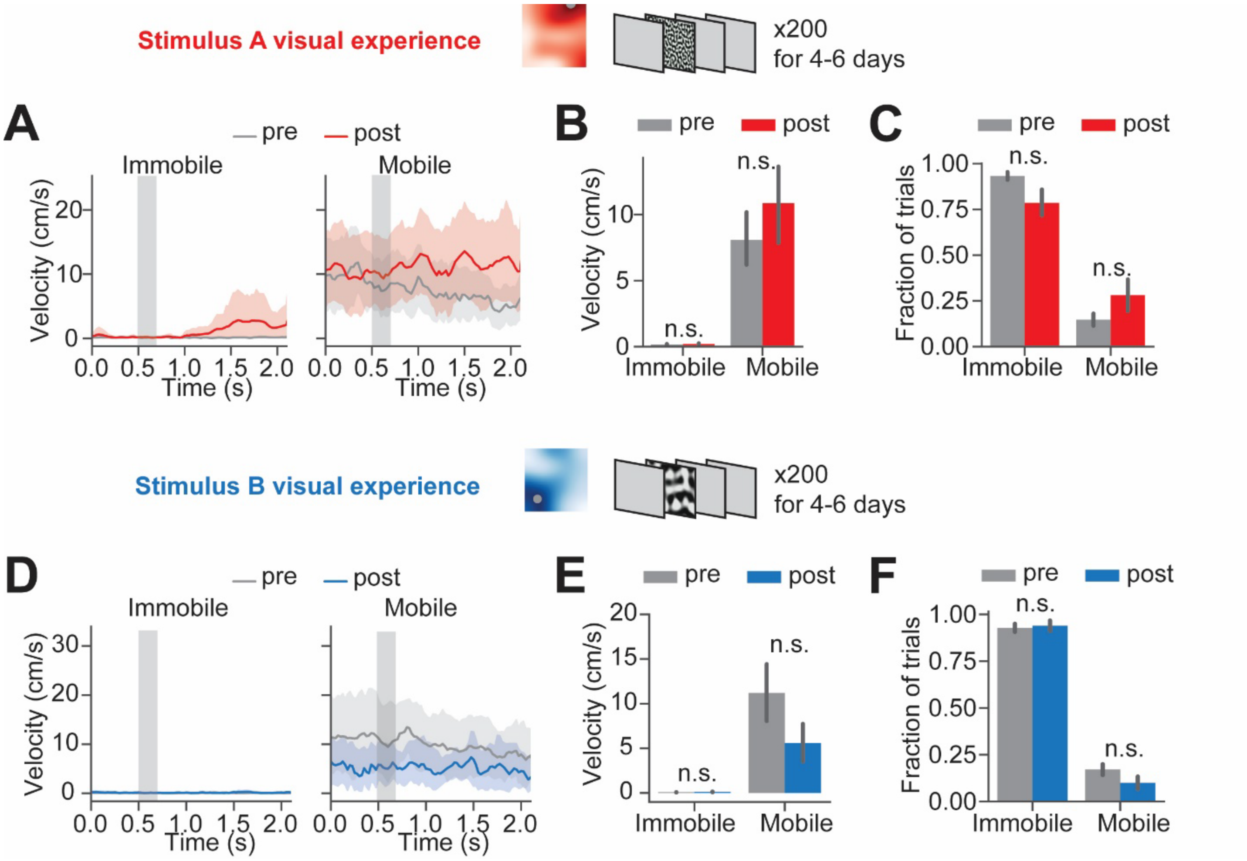

Figure S7. The fraction of the mobile trials and the locomoting velocity did not change after the entrainment of stimulus A or B. Related to Figure 1.

(A) Trial averaged locomoting velocity plotted over time before and after the entrainment that maximally induced response in LM. The gray shade represented the visual stimulation time window. Trials with mean velocity within 0.5 – 1 s larger than 0.5 cm/s are defined as mobile trials, otherwise immobile trials. Data were presented as mean ± 68% CI.

(B) The mean velocity within 0.5 – 1 s before and after the entrainment that maximally induced response in LM plotted in bar plots. Data were presented as mean ± 68% CI. Mann-Whitney U test with FDR-BH correction.

(C) The fractions of immobile and mobile trials before and after the entrainment that maximally induced response in LM plotted in bar plots. Data were presented as mean ± 68% CI. Mann-Whitney U test with FDR-BH correction.

(D) Trial averaged locomoting velocity plotted over time before and after the entrainment that maximally induced response in AL. The gray shade represented the visual stimulation time window. Trials with mean velocity within 0.5 – 1 s larger than 0.5 cm/s are defined as mobile trials, otherwise immobile trials. Data were presented as mean ± 68% CI.

(E) The mean velocity within 0.5 – 1 s before and after the entrainment that maximally induced response in AL plotted in bar plots. Data were presented as mean ± 68% CI. Mann-Whitney U test with FDR-BH correction.

(F) The fractions of immobile and mobile trials before and after the entrainment that maximally induced response in AL plotted in bar plots. Data were presented as mean ± 68% CI. Mann-Whitney U test with FDR-BH correction.

*-$p<0.05$, **-$p<0.01$, ***-$p<0.001$, n.s.-$p>0.05$.

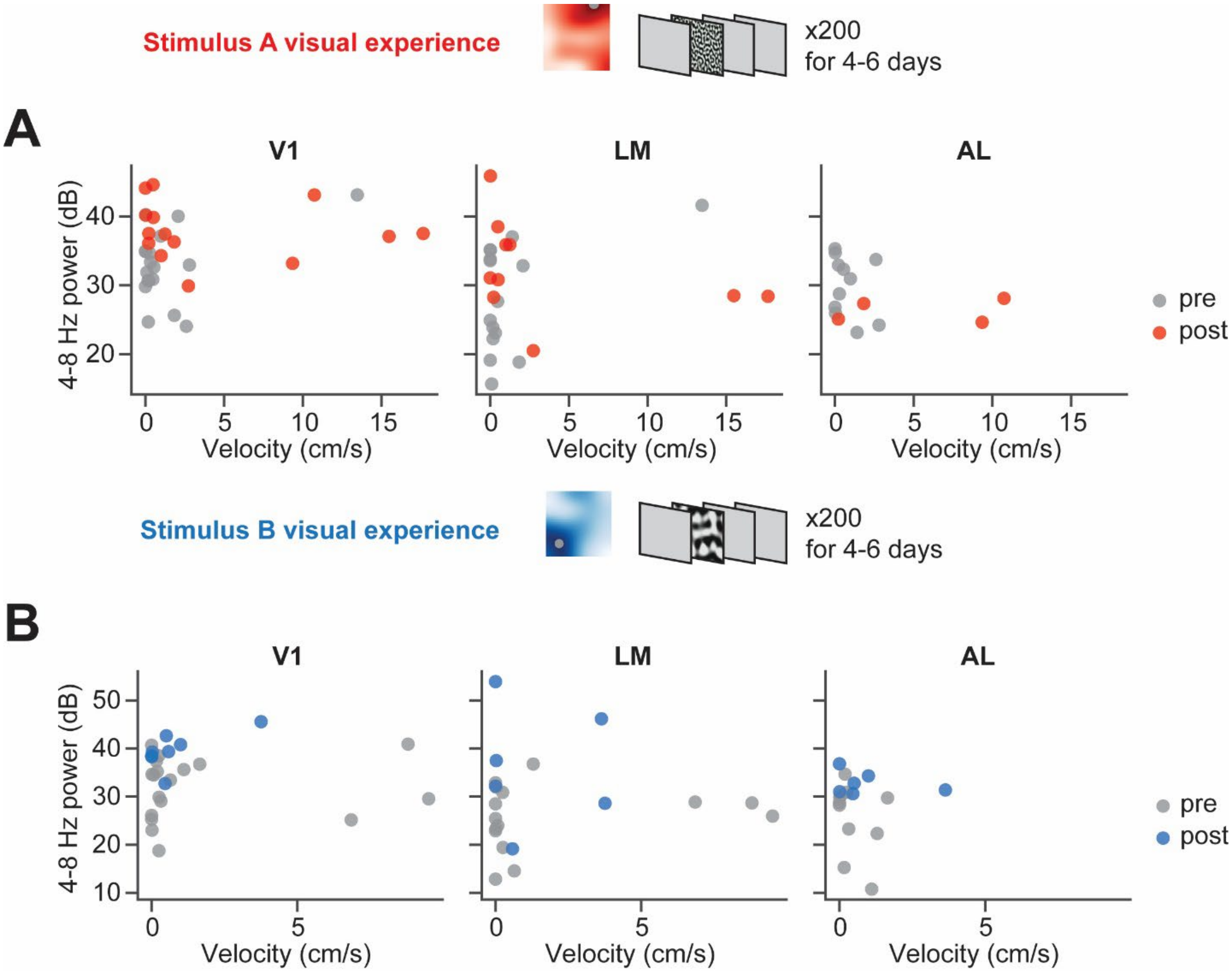

Figure S8. 4-8 Hz power and locomoting velocity do not show correlations. Related to Figure 1.

(A) Scatterplot comparison between 4-8 Hz power of trial averaged LFPs within 700 ms after the stimulus onset and trial averaged locomoting velocity within 500 ms after the stimulus onset, before and after the entrainment that maximally induced response in LM. V1: pre: N=18 mice, post: N=14 mice; LM: pre: n=15 mice, post: n=10 mice; AL: pre: n=11 mice, post: N=4 mice.

(B) Scatterplot comparison between 4-8 Hz power of trial averaged LFPs within 700 ms after the stimulus onset and trial averaged locomoting velocity within 500 ms after the stimulus onset, before and after the entrainment that maximally induced response in AL. V1: pre:

n=18 mice, post: n=8 mice; LM: pre: n=15 mice, post: n=6 mice; AL: pre: n=11 mice, post: n=6 mice.

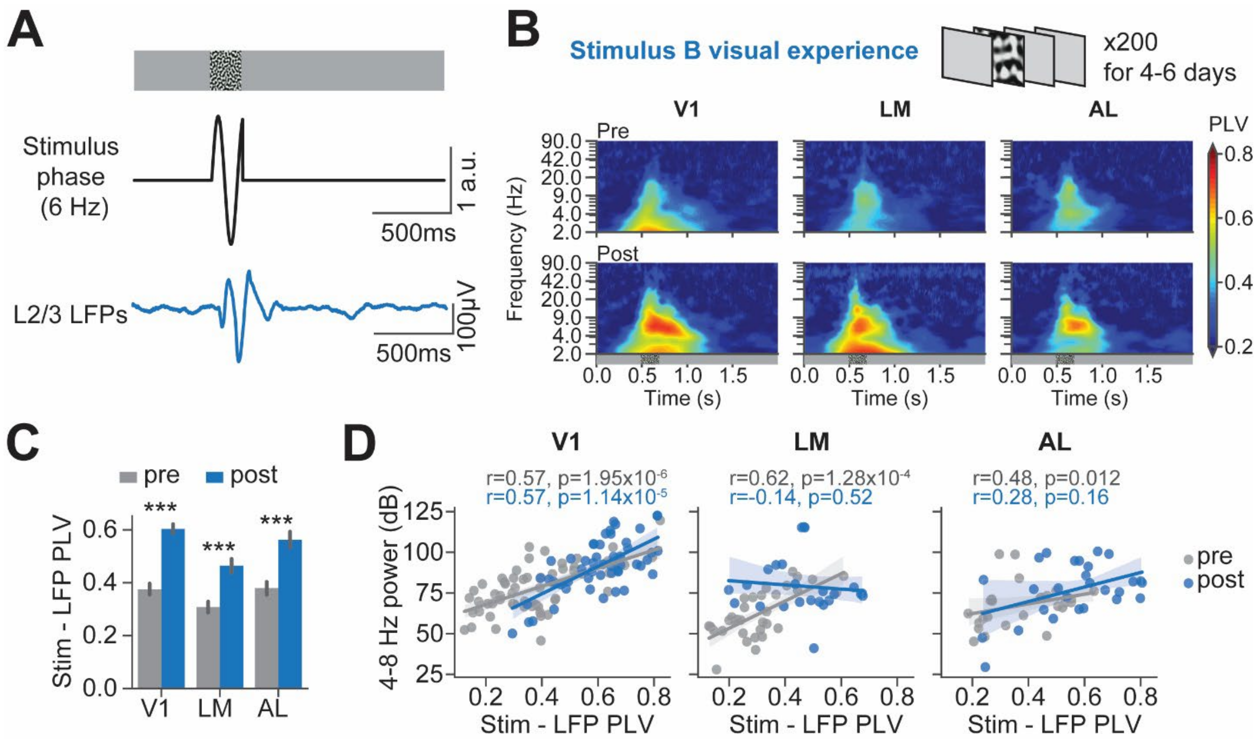

Figure S9. Inter-areal 4-8 Hz phase-locking was positively correlated with the phase-locking between visual stimulus phases and LFPs, yet V1-AL phase-locking was stronger than V1-LM phase-locking after the entrainment of SF and TF that maximally induced responses in AL. Related to Figure 1.

(A) Phase-locking analysis was performed between 6 Hz sinusoidal phases and layer 2/3 LFPs.

(B) Heatmaps showing the average phase-locking values (PLVs) between 6 Hz sinusoidal phases and layer 2/3 LFPs.

(C) Bar plots showing the median PLVs within 700 ms after the visual stimulus onset between 4-8 Hz. V1: T=8.12, $p=8.06\times10^{-13}$, n_{pre}= 60 LFPs, 30 mice, n_{post}= 51 LFPs, 27 mice; LM: T=4.51, $p=4.48\times10^{--5}$, n_{pre}= 33 LFPs, 11 mice, n_{post}=27 LFPs, 9 mice; AL: T=4.42, $p=4.98\times10^{--5}$, n_{pre}=27 LFPs, 9 mice, n_{post}=27 LFPs, 9 mice. Unpaired t-tests.

(D) Scatter plots showing the 4-8 Hz stimulus – LFP (Stim – LFP) PLVs versus 4-8 Hz power of LFPs. Spearman R values and p values shown in the inset.

*-$p<0.05$, **-$p<0.01$, ***-$p<0.001$, n.s.-$p>0.05$.

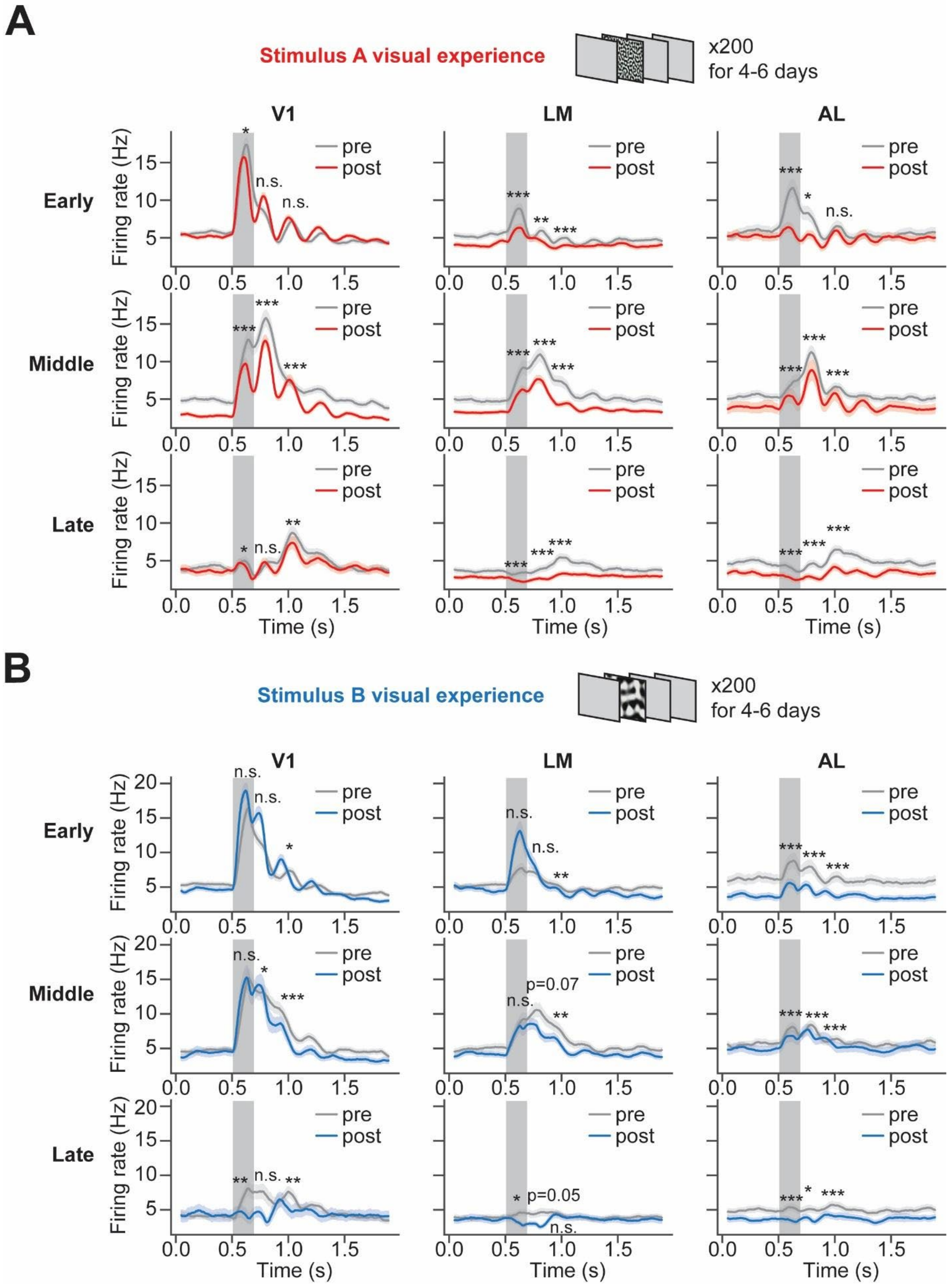
A
Stimulus A visual experience
x200
for 4-6 days
V1
LM
AL
Early
Middle
Late
Firing rate (Hz)
pre
post
n.s.
*
**
***
Time (s)
B
Stimulus B visual experience
x200
for 4-6 days
p=0.07
p=0.05

Figure S10. Firing rates of early-, middle-, late- firing units before and after visual experience. Related to Figure 4.

(A) Averaged firing rates of early-, middle-, late- firing units before and after stimulus A visual experience. The visual stimulation time window was indicated by the gray shaded area. Data were presented as mean ± 68% CI. See extended table 18 for detailed statistics.

(B) Averaged firing rates of early-, middle-, late- firing units before and after stimulus B visual experience. The visual stimulation time window was indicated by the gray shaded area. Data were presented as mean ± 68% CI. See extended table 19 for detailed statistics.

*-$p<0.05$, **-$p<0.01$, ***-$p<0.001$, n.s.-$p>0.05$.

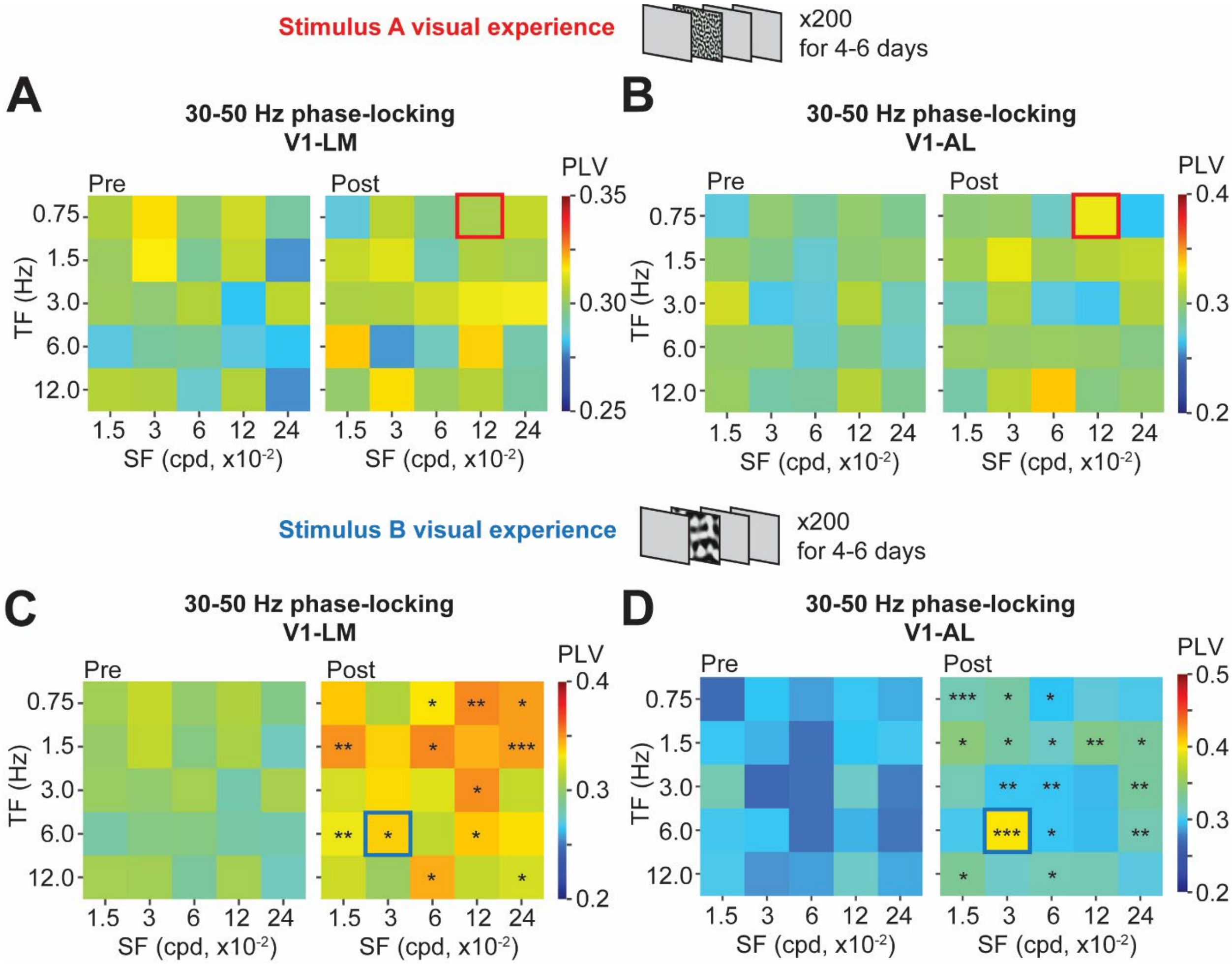

Figure S11. 30-50 Hz phase-locking did not increase after the entrainment of the SF and TF that maximally induced response in LM, but increased after the entrainment of the SF and TF that maximally induced response in AL. Related to Figure 2 and Figure 3.

(A) Median 30-50 Hz PLV values of V1-LM LFP pairs in responses to 5 SFs and 5 TFs after the entrainment of SF and TF that maximally induced responses in LM were plotted in heatmaps. Statistically significant differences between post- and pre- experience for each visual stimulus were labeled. Mann-Whitney U test with FDR-BH correction. See extended table 9 for detailed statistics.

(B) Same as (A) but for V1-AL LFPs pairs. See extended table 10 for detailed statistics.

(C) Same as (A) but for the entrainment of SF and TF that maximally induced responses in AL. See extended table 11 for detailed statistics.

(D) Same as (C) but for V1-AL LFPs pairs. See extended table 12 for detailed statistics.

*-$p<0.05$, **-$p<0.01$, ***-$p<0.001$, n.s.-$p>0.05$.

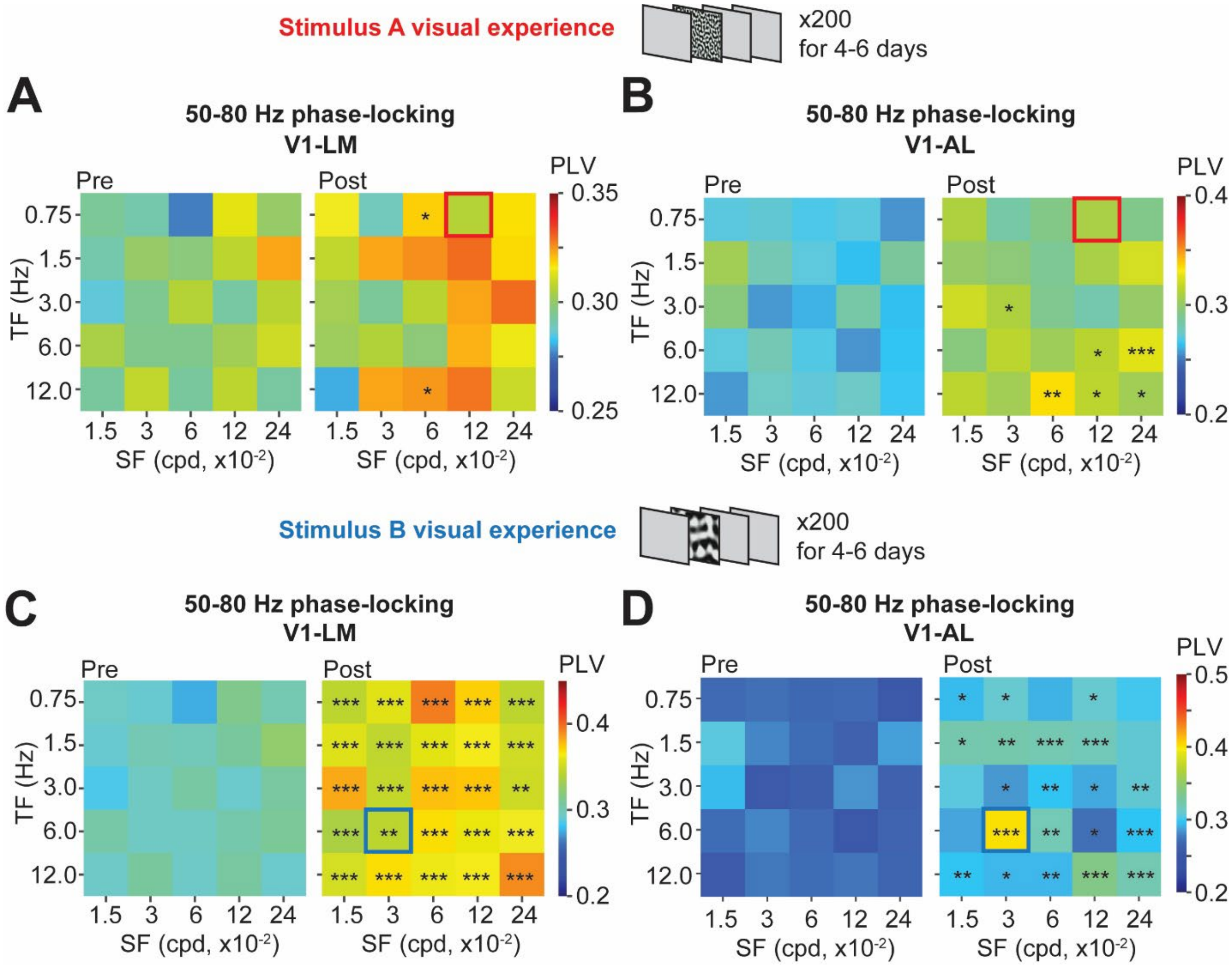

Figure S12. 50-80 Hz phase-locking did not increase after the entrainment of the stimulus that maximally induced response in LM, but increased after the entrainment of the stimulus that maximally induced response in AL. Related to Figure 2 and Figure 3.

(A) Median 50-80 Hz PLV values of V1-LM LFP pairs in responses to 5 SFs and 5 TFs after the entrainment of SF and TF that maximally induced responses in LM were plotted in heatmaps. Statistically significant differences between post- and pre- experience for each visual stimulus were labeled. Mann-Whitney U test with FDR-BH correction. See extended table 13 for detailed statistics.

(B) Same as (A) but for V1-AL LFPs pairs. See extended table 14 for detailed statistics.

(C) Same as (A) but for the entrainment of SF and TF that maximally induced responses in AL. See extended table 15 for detailed statistics.

(D) Same as (C) but for V1-AL LFPs pairs. See extended table 16 for detailed statistics.

*-p<0.05, **-p<0.01, ***-p<0.001, n.s.-p>0.05.

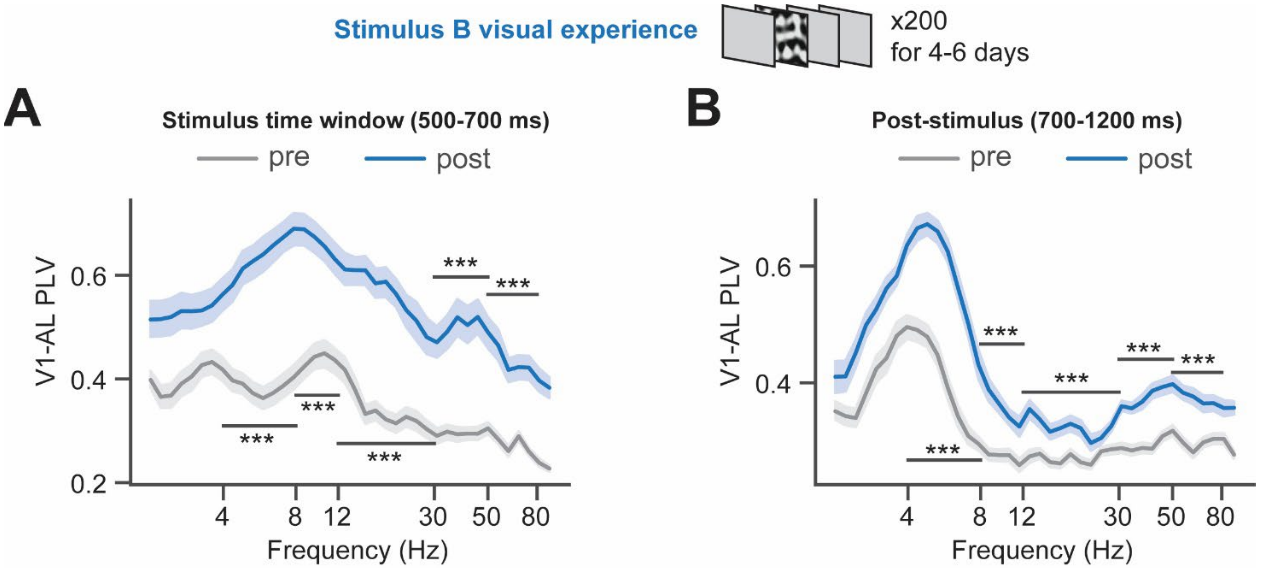

Figure S13. V1-AL phase-locking values during the stimulus time window and post-stimulus time window after the entrainment of SF and TF that maximally induced response in AL. Related to Figure 3.

(A) V1-AL PLVs during the stimulus time window (median values within 500-700 ms). n_{pre}=81 LFP pairs, 9 mice; n_{post}=54 LFP pairs, 6 mice. 4-8 Hz pre vs. post: U=731, p=2.68x10^{-10}; 8-12 Hz pre vs. post: U=1028, p=2.45x10^{-7}; 12-30 Hz pre vs. post: U=749, p=2.68x10^{-10}; 30-50 Hz pre vs. post: U=1121, p=1.7x10^{-6}; 50-80 Hz pre vs. post: U=1000, p=1.65x10^{-7}. Mann-Whitney U tests with FDR-BH correction.

(B) V1-AL PLVs during the post-stimulus time window (median values within 700-1200 ms). n_{pre}=81 LFP pairs, 9 mice; n_{post}=54 LFP pairs, 6 mice. 4-8 Hz pre vs. post: U=666, p=4.27x10^{-11}; 8-12 Hz pre vs. post: U=1333, p=1.3x10^{-4}; 12-30 Hz pre vs. post: U=1315, p=1.1x10^{-4}; 30-50 Hz pre vs. post: U=1032, p=5.39x10^{-7}; 50-80 Hz pre vs. post: U=1185, p=1.14x10^{-5}. Mann-Whitney U tests with FDR-BH correction.

*-p<0.05, **-p<0.01, ***-p<0.001, n.s.-p>0.05.

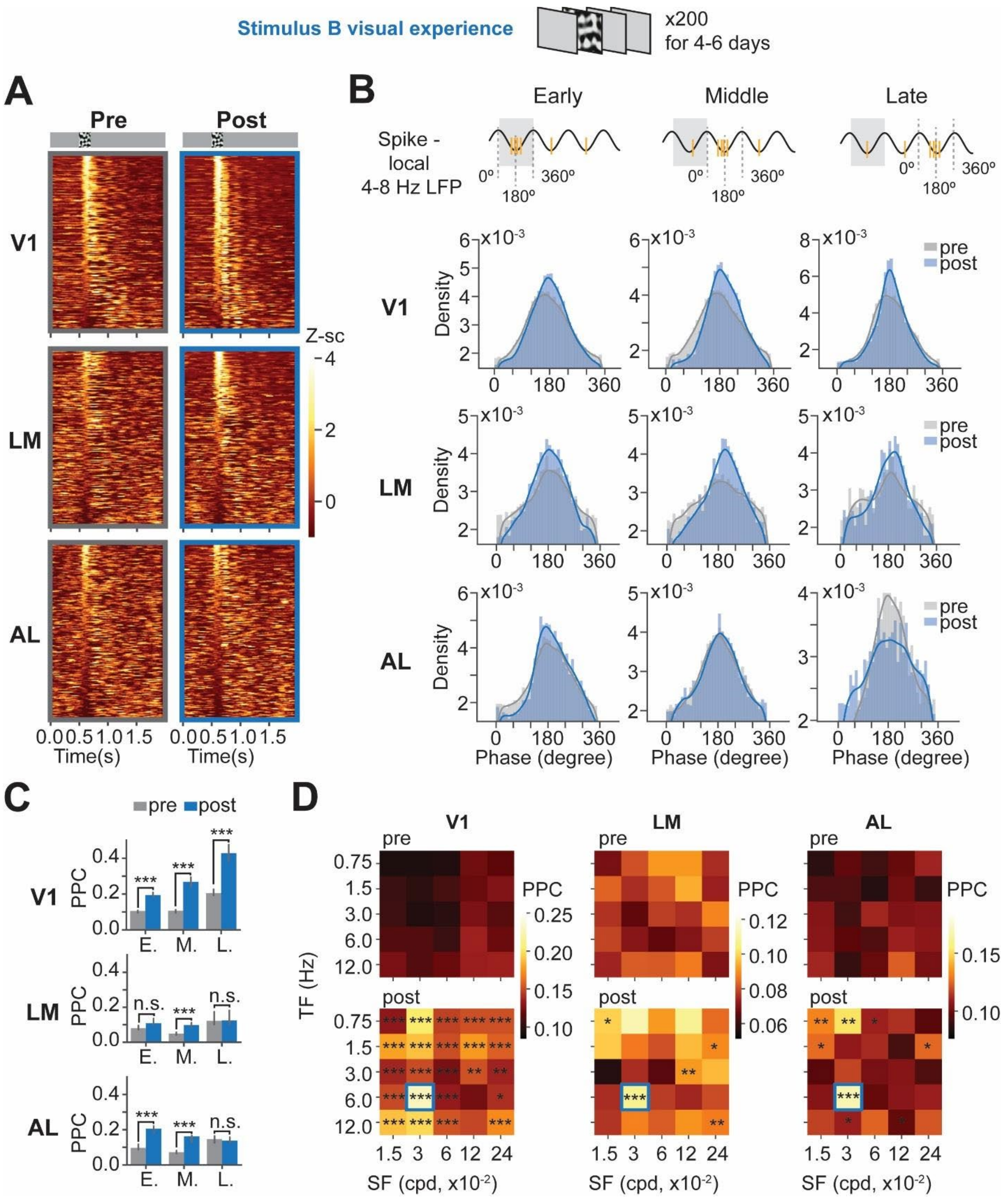

Stimulus B visual experience
x200
for 4-6 days
A
Pre
Post
V1
LM
AL
Z-sc
4
2
0
0.0 0.5 1.0 1.5
Time(s)
B
Early
Middle
Late
Spike - local 4-8 Hz LFP
0º
180º
360º
Density
pre
post
Phase (degree)
C
PPC
E.
M.
L.
n.s.
D
TF (Hz)
SF (cpd, x10-2)
PPC

Figure S14. Single units in V1, LM, and AL spiked at more consistent 4-8 Hz phases after the entrainment of SF and TF that maximally induced response in AL. Related to Figure 4.

(A) Units' firing rate z-scores over time were plotted in heatmaps. V1: n_{pre}: 921 units, 15 mice, n_{post}: 916 units, 14 mice; LM: n_{pre}: 666 units, 9 mice, n_{post}: 778 units, 11 mice; AL: n_{pre}: 711 mice, 11 mice, n_{post}: 657 units, 9 mice.

(B) Units were grouped into early-, middle-, and late- firing units based on the time windows of their peak firing rate z-score. 4-8 Hz spike phases of phase selective units in relation to local LFPs were plotted in density plots.

(C) Pairwise phase consistency values (PPC, calculated using spike phases within 700 ms post stimulus onset) of 4-8 Hz phase-selective units were plotted in bar plots. V1: Pre: N_{early}: 391 units, n_{middle}: 258 units, n_{late}: 127 units, 18 mice, Post: n_{early}: 439 units, n_{middle}: 141 units, n_{late}: 75 units, 14 mice; LM: Pre: n_{early}: 92 units, n_{middle}: 140 units, n_{late}: 39 units, 14 mice, Post: n_{early}: 117 units, n_{middle}: 96 units, n_{late}: 49 units, 9 mice; AL: Pre: n_{early}: 134 units, n_{middle}: 120 units, n_{late}: 69 units, 12 mice, Post: n_{early}: 102 units, n_{middle}: 105 units, n_{late}: 62 units, 9 mice. Data are represented as median ± 68% CI. V1: early: $p=1.5x10^{-17}$, middle: $p=1.79x10^{-11}$, late: $p=1.77x10^{-6}$; LM: early: p=0.067, middle: $p=1.68x10^{-3}$, late: p=0.899; AL: early: $p=3.87x10^{-7}$, middle: $p=6.29x10^{-5}$, late: p=0.848, Mann-Whitney U test with FDR-BH correction.

(D) Averaged 4-8 Hz PPCs were plotted in heatmaps. The entrained stimulus was indicated by the blue square. The asterisks represented statistical significances in PPCs between pre- and post- visual experience. See extended table 8 for detailed statistics.

*-p<0.05, **-p<0.01, ***-p<0.001, n.s.-p>0.05.

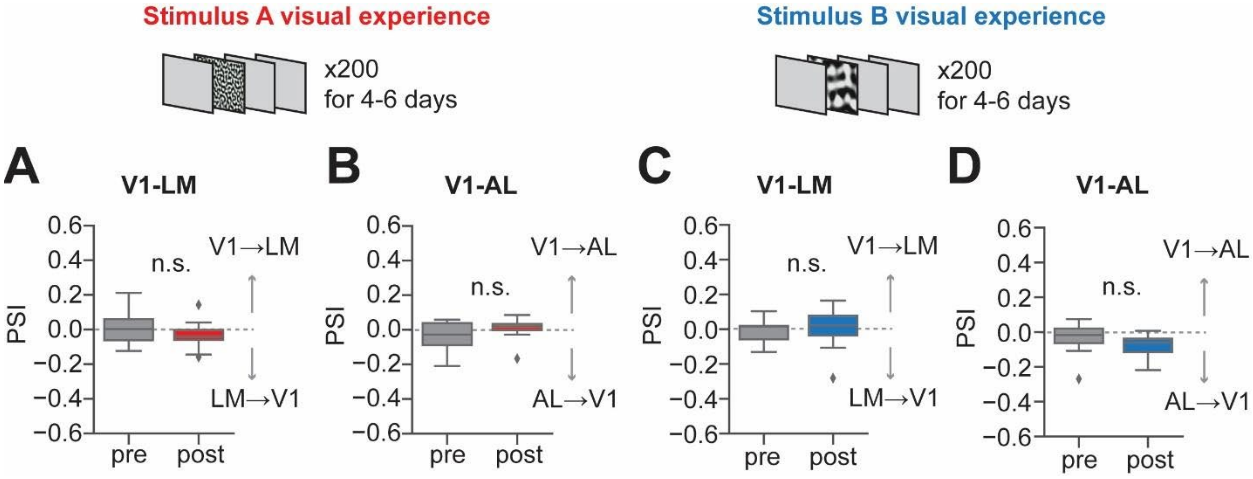

Figure S15. Phase slope indices of V1-LM LFP pairs and V1-AL LFP pairs after the visual experience. Related to Figure 5.

(A) 4-8 Hz phase slope indices (PSI) between V1 and LM were plotted in box plots. n_{pre}=11 mice, n_{post}=15 mice, p=0.426, Mann-Whitney U test.

(B) 4-8 Hz phase slope indices (PSI) between V1 and AL were plotted in box plots. n_{pre}=9 mice, n_{post}=8 mice, p=0.846, Mann-Whitney U test.

(C) 4-8 Hz phase slope indices (PSI) between V1 and LM were plotted in box plots. n_{pre}=11 mice, n_{post}=8 mice, p=0.984, Mann-Whitney U test.

(D) 4-8 Hz phase slope indices (PSI) between V1 and LM were plotted in box plots. n_{pre}=9 mice, n_{post}=9 mice, p=0.315, Mann-Whitney U test.

*-p<0.05, **-p<0.01, ***-p<0.001, n.s.-p>0.05.

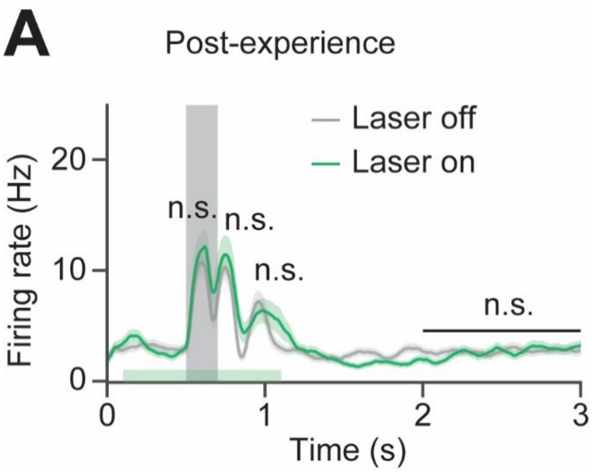

Figure S16. V1 unit firing rates with and without inactivating LM after the visual experience. Related to Figure 6.

(A) V1 unit firing rates with and without inactivating LM after the visual experience. The gray shade represents the visual stimulation time window. The green shade represents the inactivation time window. Data are presented as mean ± S.E.M. n = 134 units, 5 mice. The oscillation peak firing rates with and without inactivating LM were compared by Mann-Whitney U tests. The median firing rates between 2-3 s with and without inactivating LM were compared by Mann-Whitney U test.

n.s. – not significant.

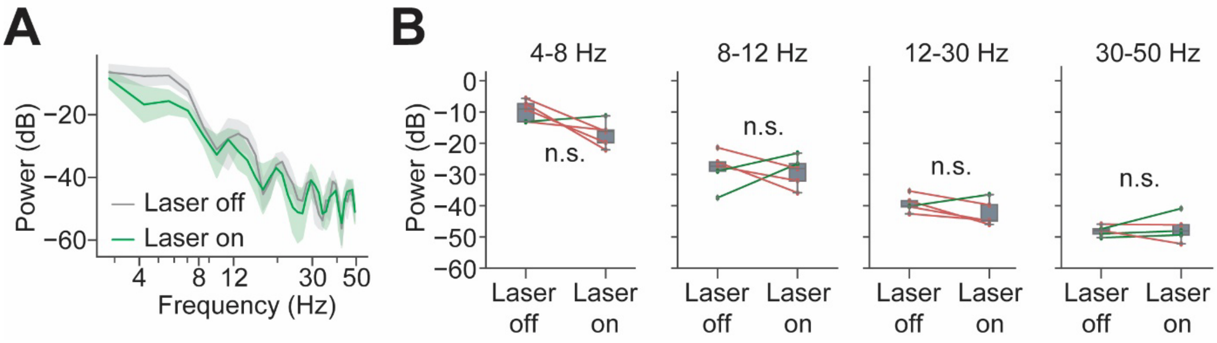

Figure S17. Power spectrum of multi-unit firing rates in V1. Related to Figure 6.

(A) Power spectrum of multi-unit activity of V1 superficial layer and layer 4 units. Data are presented as mean ± S.E.M. n = 5 mice.

(B) Mean power of V1 multi-unit firing rates within each frequency band. n = 5 mice. Wilcoxon signed-rank test.

n.s. – not significant.

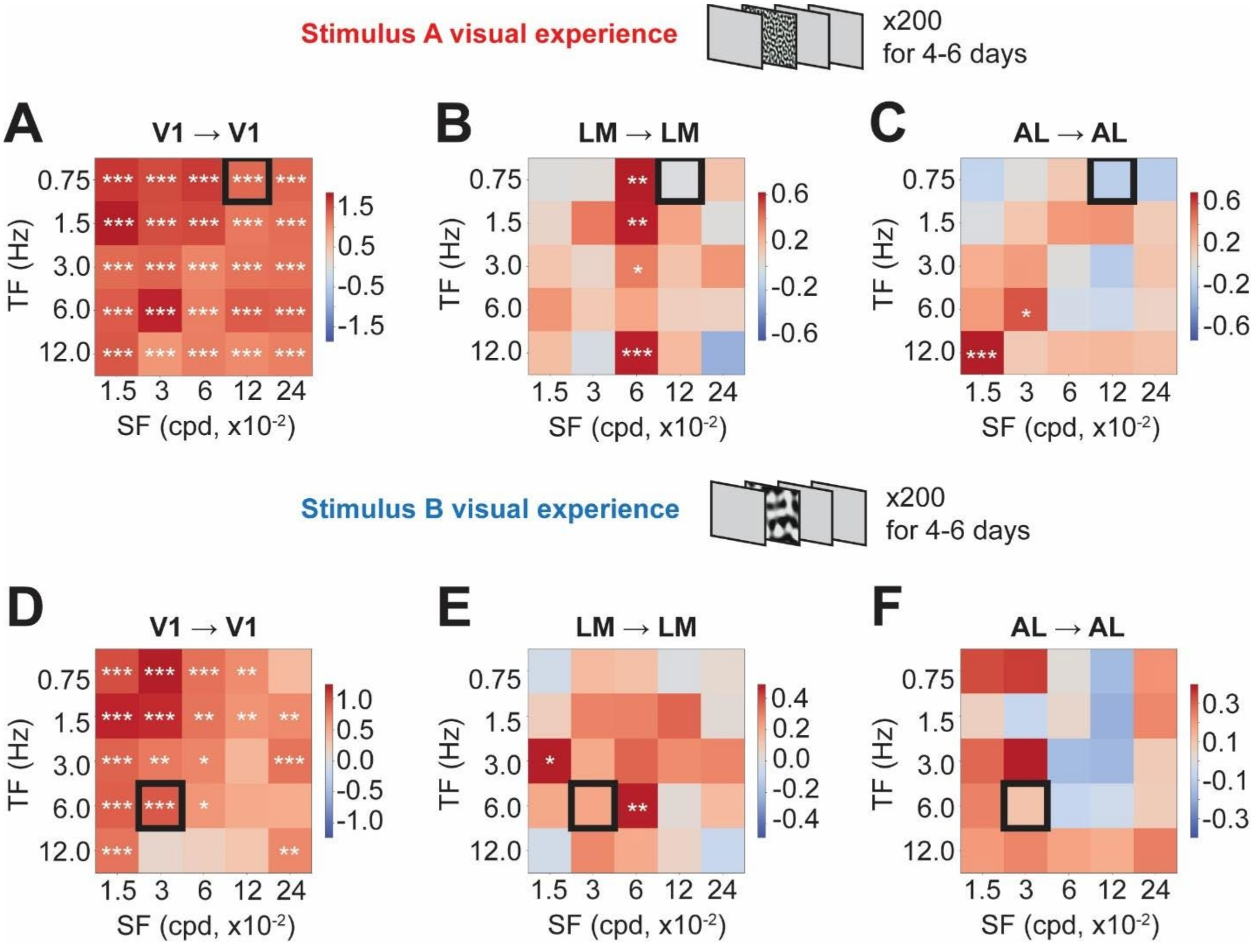

Figure S18. Functional connectivity changes within each area after the visual experience. Related to Figure 5.

(A) Differences in V1-to-V1 functional connectivity between post- and pre- stimulus A visual experience were plotted in a heatmap. The entrained SF/TF combination was indicated by the black circle.

(B) Differences in LM-to-LM functional connectivity between post- and pre- stimulus A visual experience were plotted in a heatmap. The entrained SF/TF combination was indicated by the black circle.

(C) Differences in AL-to-AL functional connectivity between post- and pre- stimulus A visual experience were plotted in a heatmap. The entrained SF/TF combination was indicated by the black circle.

(D) Differences in V1-to-V1 functional connectivity between post- and pre- stimulus B visual experience were plotted in a heatmap. The entrained SF/TF combination was indicated by the black circle.

(E) Differences in LM-to-LM functional connectivity between post- and pre- stimulus B visual experience were plotted in a heatmap. The entrained SF/TF combination was indicated by the black circle.

(F) Differences in AL-to-AL functional connectivity between post- and pre- stimulus B visual experience were plotted in a heatmap. The entrained SF/TF combination was indicated by the black circle.

*-$p<0.05$, **-$p<0.01$, ***-$p<0.001$, n.s.-$p>0.05$.

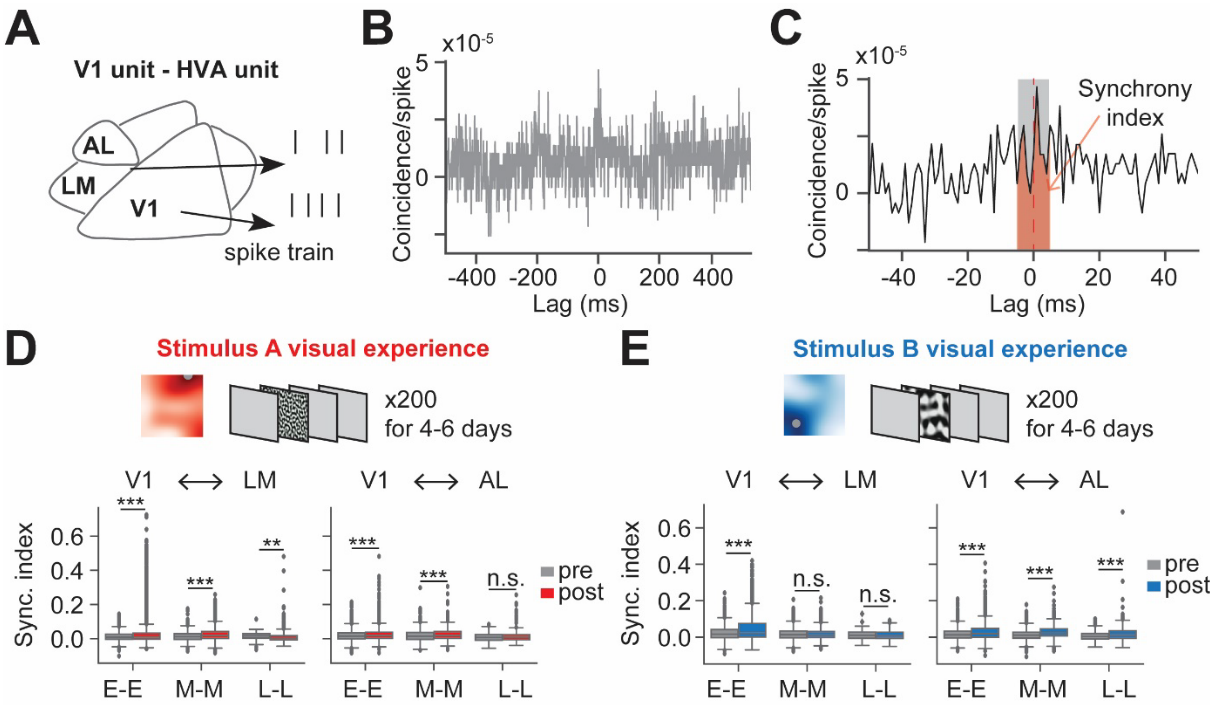

Figure S19. Cross-correlation between unit spike trains from V1 and HVA increased after the visual experience. Related to Figure 5.

(A) Jitter-corrected spike train cross-correlations were calculated for units that were simultaneously recorded in V1 and LM, or in V1 and AL.

(B) A representative spike train cross-correlation (after firing rate correction and jitter correction) of a V1-LM unit pair.

(C) The cross-correlation of the unit pair in (B) showed a high correlation peak (>2 std. of ±100 ms from 0 ms) within ±5 ms time lag. The area under the curve within the ±5 ms from 0 ms was quantified as the synchrony index for a unit pair. Unit pairs that showed such a peak in response to at least one visual stimulus were selected for further analyses.

(D) Synchrony indices of highly correlated unit pairs in response to the entrained SF and TF that maximally induced response in LM were plotted in boxplots. Synchrony indices of early-early (E-E), middle-middle (M-M), late-late (L-L) unit pairs were analyzed. V1-LM: Pre: $n_{E\text{-}E}$: 2597 unit pairs, $n_{M\text{-}M}$: 1076 unit pairs, $n_{L\text{-}L}$: 91 unit pairs, 9 mice; Post: $n_{E\text{-}E}$: 5131 unit pairs, $n_{M\text{-}M}$: 2308 unit pairs, $n_{L\text{-}L}$: 309 unit pairs, 13 mice. V1-AL: Pre: $n_{E\text{-}E}$: 4283 unit pairs, $n_{M\text{-}M}$: 1330 unit

pairs, n_{L-L}: 434 unit pairs, 9 mice; Post: n_{E-E}: 2278 unit pairs, n_{M-M}: 1210 unit pairs, n_{L-L}: 304 unit pairs, 6 mice. V1-LM: early: CLES=0.537, $p=2.06 \times 10^{-7}$, middle: CLES=0.584, $p=8.19 \times 10^{-15}$, late: CLES=0.399, $p=3.37 \times 10^{-3}$; V1-AL: early: CLES=0.540, $p=3.93 \times 10^{-7}$, middle: CLES=0.554, $p=3.04 \times 10^{-6}$, late: CLES=0.504, p=0.869, Mann-Whitney U test with FDR-BH correction.

(E) Synchrony indices of highly correlated unit pairs in response to the entrained SF and TF that maximally induced response in LM were plotted in boxplots. Synchrony indices of early-early (E-E), middle-middle (M-M), late-late (L-L) unit pairs were analyzed. V1-LM: Pre: n_{E-E}: 1958 unit pairs, n_{M-M}: 1833 unit pairs, n_{L-L}: 120 unit pairs, 9 mice; Post: n_{E-E}: 2604 unit pairs, n_{M-M}: 818 unit pairs, n_{L-L}: 221 unit pairs, 6 mice. V1-AL: Pre: n_{E-E}: 3544 unit pairs, n_{M-M}: 1420 unit pairs, n_{L-L}: 363 unit pairs, 9 mice; Post: n_{E-E}: 1428 unit pairs, n_{M-M}: 562 unit pairs, n_{L-L}: 170 unit pairs, 6 mice. V1-LM: early: CLES=0.580, $p=6.93 \times 10^{-20}$, middle: CLES=0.499, p=0.957, late: CLES=0.499, p=0.957; V1-AL: early: CLES=0.573, $p=1.16 \times 10^{-15}$, middle: CLES=0.647, $p=7.16 \times 10^{-24}$, late: CLES=0.595, $p=3.96 \times 10^{-4}$, Mann-Whitney U test with FDR-BH correction.

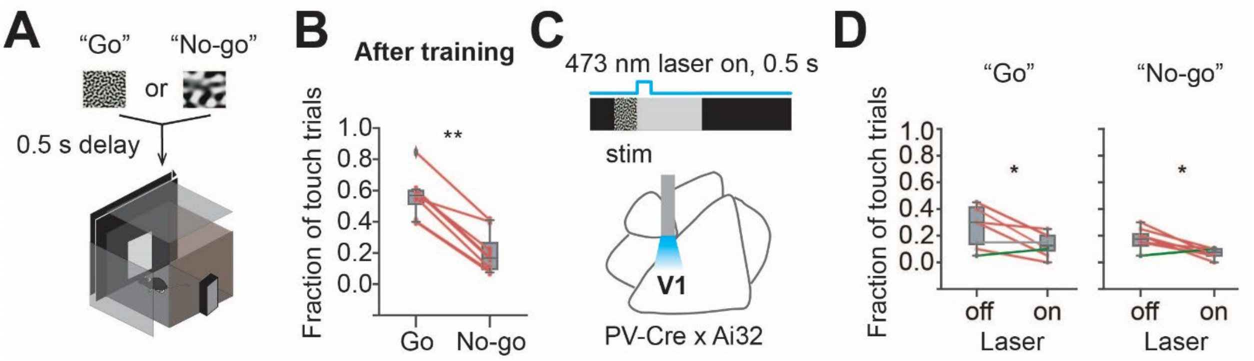

Figure S20. Optogenetic inactivation of V1 post-stimulus activity impaired visually-cued "Go" behavior. Related to Figure 7.

(A) Mice were trained to perform a visually-cued Go/No-go task using a touchscreen chamber.

(B) Fraction of touch trials out of twenty trials on training day 25. $p=7.81 \times 10^{-3}$, $n_{Go}=n_{No\text{-}go}=8$ mice, Wilcoxon signed-rank test.

(C) Following the training, bilateral V1 was inactivated by activating PV-ChR2+ neurons using 473 nm light through an optic cannula on top of V1. The laser was turned for 0.5 s following the visual stimulus before the touch response time window.

(D) The fraction of touch trials with the laser turned off and turned on were plotted. "Go" trials: $p=0.04$, $n=8$ mice; "No-Go" trials: $p=0.04$, $n=8$ mice. Wilcoxon signed-rank test.

*-$p<0.05$, **-$p<0.01$.
